# Analysis of TET Expression/Activity and 5mC Oxidation during Normal and Malignant Germ Cell Development

**DOI:** 10.1371/journal.pone.0082881

**Published:** 2013-12-26

**Authors:** Daniel Nettersheim, Lukas C. Heukamp, Florian Fronhoffs, Marc J. Grewe, Natalie Haas, Anke Waha, Friedemann Honecker, Andreas Waha, Glen Kristiansen, Hubert Schorle

**Affiliations:** 1 Institute of Pathology, Department of Developmental Pathology, University Hospital, Bonn, Germany; 2 Institute of Pathology, University Hospital, Bonn, Germany; 3 Institute of Neuropathology, University Hospital, Bonn, Germany; 4 Department of Oncology, Haematology and Bone Marrow Transplantation with Section Pneumology, Hubertus Wald-Tumorzentrum, University Medical Center Hamburg-Eppendorf, Hamburg, Germany; Virginia Commonwealth University, United States of America

## Abstract

During mammalian development the fertilized zygote and primordial germ cells lose their DNA methylation within one cell cycle leading to the concept of active DNA demethylation. Recent studies identified the TET hydroxylases as key enzymes responsible for active DNA demethylation, catalyzing the oxidation of 5-methylcytosine to 5-hydroxymethylcytosine. Further oxidation and activation of the base excision repair mechanism leads to replacement of a modified cytosine by an unmodified one. In this study, we analyzed the expression/activity of TET1-3 and screened for the presence of 5mC oxidation products in adult human testis and in germ cell cancers. By analyzing human testis sections, we show that levels of 5-hydroxymethylcytosine, 5-formylcytosine and 5-carboxylcytosine are decreasing as spermatogenesis proceeds, while 5-methylcytosine levels remain constant. These data indicate that during spermatogenesis active DNA demethylation becomes downregulated leading to a conservation of the methylation marks in mature sperm. We demonstrate that all carcinoma in situ and the majority of seminomas are hypomethylated and hypohydroxymethylated compared to non-seminomas. Interestingly, 5-formylcytosine and 5-carboxylcytosine were detectable in all germ cell cancer entities analyzed, but levels did not correlate to the 5-methylcytosine or 5-hydroxymethylcytosine status. A meta-analysis of gene expression data of germ cell cancer tissues and corresponding cell lines demonstrates high expression of *TET1* and the DNA glycosylase *TDG,* suggesting that germ cell cancers utilize the oxidation pathway for active DNA demethylation. During xenograft experiments, where seminoma-like TCam-2 cells transit to an embryonal carcinoma-like state *DNMT3B* and *DNMT3L* where strongly upregulated, which correlated to increasing 5-methylcytosine levels. Additionally, 5-hydroxymethylcytosine levels were elevated, demonstrating that de novo methylation and active demethylation accompanies this transition process. Finally, mutations of *IDH1* (*IDH1* ^R132^) and *IDH2* (*IDH2* ^R172^) leading to production of the TET inhibiting oncometabolite 2-hydroxyglutarate in germ cell cancer cell lines were not detected.

## Introduction

In the past decade, epigenetic profiles of various cell types and tissues were analyzed with great ambition. In general, methylation of cytosines within CpG dinucleotides of promoter regions leads to epigenetic gene silencing. The protein family of DNA methyltransferases (DNMTs) catalyzes the methylation of cytosines. DNMT1 is a maintenance methylase that remethylates the newly synthesized DNA strand during replication, requiring the hemimethylated parental strand as a blueprint. The DNMTs 3A and 3B are able to methylate DNA de novo in cooperation with the cofactor DNMT3L. In contrast to this, the question how DNA can become demethylated is only poorly understood. The observation that the fertilized zygote and primordial germ cells (PGCs) lose methylation within one cell cycle led to the hypothesis that these cells are able to actively remove methylation marks in their genome [1] [2] [3] [4] [5]. Several publications of the last years could demonstrate that oxidation of 5-methylcytosine (5mC) to 5-hydroxymethylcytosine (5hmC) by the ten-eleven-translocation (TET) proteins is a key process of active DNA demethylation (ADD) [6] [7]. It is hypothesized that 5hmC is converted into a cytosine by two mechanisms – the oxidation and the deamination pathway [8]. The oxidation pathway involves further oxidation steps of 5hmC to 5-formylcytosine (5fC) and 5-carboxylcytosine (5caC) by the TET proteins. 5fC and 5caC can be replaced by an unmodified cytosine by the base excision repair (BER) pathway, involving the enzyme thymine DNA glycosylase (TDG) [9] [10]. Nevertheless, it is also suggested that 5fC and 5caC might become deformylated or decarboxylated by so far unidentified deformylases or decarboxylases, eventually resulting in replacement of modified cytosines by unmodified cytosines [8]. The deamination pathway involves deaminases of the AID or APOBEC family to deaminate 5mC or 5hmC to thymidine or 5-hydroxymethyl-uracil, respectively. Both deamination products are thought to be replaced by cytosines by the BER pathway, involving TDG, MBD4 or the single strand selective monofunctional uracil DNA glycosylase (SMUG1) [8].

In mice, it has been shown that oxidation of 5mC to 5hmC is mainly catalyzed by Tet1 [11]. *Tet1* knock-down resulted in an increase in global and Nanog promotor specific DNA methylation, while 5hmC levels become reduced [12]. Furthermore, TET2 and TET3 are able to catalyze the same reactions as TET1, but TET3 has only been reported to be active in ADD of the male pronucleus at zygote stage [12] [13].

Testicular type II germ cell cancers (GCC) arise from a common precursor lesion, the carcinoma in situ (CIS) [14] [15]. CIS cells are thought to arise from a PGC, which is arrested in development. GCCs can be subdivided into two groups – the seminomas and the non-seminomas [15]. Seminomas are considered to be the default developmental pathway of CIS cells [16]. The non-seminomas comprise of the pluripotent undifferentiated embryonal carcinomas (EC) and the more differentiated teratomas, yolk-sac tumors and choriocarcinomas.

Recently, it has been demonstrated that mutations of the isocitrate dehydrogenases (*IDH*) 1 and 2 genes (*IDH1* ^R132^, *IDH2^R172^*) in glioblastomas, diffuse astrocytic and oligodendroglial tumors are associated with the production of the oncometabolite 2-hydroxyglutarate and decreased levels of alpha-ketoglutarate, leading to inhibition of alpha-ketoglutarate-dependent oxygenases, which are involved in DNA and histone tail demethylation [17] [18] [19] [20]. Additionally, mutations of the IDH genes are associated with increased DNA methylation at CpG islands [21]. Since TET enzymes require alpha-ketoglutarate and oxygen [22], mutations of *IDH1* and *IDH2* might have an impact on ADD processes in GCCs.

Here we present a comprehensive study of human adult germ cells, GCCs and their respective cell lines with regard to ADD pathway keyplayers, like TET1-3 and global cytosine modifications. Further, we asked if changes in ADD keyplayer expression and cytosine modification levels (5mC/5hmC) accompany the transition from the seminoma-like cell line TCam-2 into an EC-like state in vivo.

Our results suggest that in spermatogonia of the adult testis oxidation of 5mC to 5hmC and further to 5fC as well as 5caC occurs. While 5mC levels are maintained until post-meiotic stages of spermatogenesis, 5hmC, 5fC and 5caC levels gradually decrease during mitosis/meiosis. By extending our analysis to human type II GCCs, we found the majority of CIS and seminoma cells to be hypomethylated and hypohydroxymethylated, while non-seminomas displayed high 5mC and 5hmC levels, demonstrating correlation between 5mC and 5hmC. Interestingly, 5fC and 5caC was detectable in all analysed GCCs, including hypomethylated and hypohydroxymethylated specimen. Quantification of 5mC, 5hmC and 5fC levels in GCC cell lines as well as qRT-PCR/cDNA microarray analysis of GCC cell lines and tissues suggest that GCCs preferentially utilize the oxidation pathway for ADD, involving *TET1*. Furthermore, we could find no mutation of the *IDH1* (*IDH1* ^R132^) and *IDH2* (*IDH2* ^R172^) genes in GCC cell lines, which are associated with aberrant production of the TET inhibiting oncometabolite 2-hydroxyglutarate. Finally, xenograft experiments demonstrated that the transition of seminomatous TCam-2 cells to an EC-like state is accompanied by strong upregulation of *DNMT3B*, *DNMT3L* and an increase in 5mC suggesting de novo methylation. Furthermore, increasing 5hmC levels suggest an ADD process during the in vivo transition.

## Materials and Methods

### Ethics statement

The ethics commitee of the Rheinische Friedrich-Wilhelms-Universität Bonn approved the analyses of formalin fixed, paraffin-embedded type-II germ cell cancer tissues in context of this study. No personal patient data will be collected or stored. Written permission to use the tissue for scientific purposes was obtained from the patients and was approved by the ‚Ethik-Kommission für klinische Versuche am Menschen und epidemiologische Forschung mit personenbezogenen Daten der Medizinischen Fakultät der Rheinischen Friedrich-Wilhelms-Universität Bonn' (The ethics committee for clinical trials on humans and epidemiological research with patient-related data of the medical faculty of the Rheinischen-Friedrich-Wilhelms-University Bonn). All animal experiments were conducted according to the German law of animal protection and in agreement with the approval of the local institutional animal care committees (Landesamt für Natur, Umwelt und Verbraucherschutz, North Rhine-Westphalia (approval ID: #8.87–50.10.31.08.238). The experiments were conducted in accordance with the ‚International Guiding Principles for Biomedical Research Involving Animals' as announced by the ‚Society for the Study of Reproduction'.

### Cell culture

TCam-2 and 1411HP cells were grown in RPMI supplemented with 10% fetal calf serum (FCS) (PAA, Pasching, Austria), 1% Penicillin/streptomycin (P/S) (PAN, Aidenbach, Germany), 200 mM L-Glutamine (PAN, Aidenbach, Germany). The cell lines 2102EP, NCCIT, 833KE, H12.1, 1777N, GCT27, NT2/D1, JEG-3 and JAR were grown in DMEM medium (10% FCS, 1% Penicillin/Streptomycin, 200 mM L-Glutamine) at 37°C and 5% CO_2_. TCam-2 [23] cells were kindly provided by Dr. Janet Shipley (Institute of Cancer Research, Sutton, United Kingdom). 2102EP [24], NT2/D1 [25] and NCCIT [26] cells were provided by Prof. Dr. Leendert Looijenga (Erasmus MC, Daniel den Hoed Cancer Center, Josephine Nefkens Institute, Rotterdam, Netherlands), 833KE [27] cells were provided by PD Dr. Beate Köberle (KIT, Karlsruhe, Germany). H12.1 [28], GCT27 [29], 1777N [27] and 1411HP [30] were kindly provided by Dr. Peter Andrews (Univeristy of Sheffield, United Kingdom) via Dr. Thomas Müller (Departement of Internal Medicine IV, Oncology and Hematology, Martin-Luther-University of Halle Wittenberg, Halle, Germany). JAR (ATCC HTB-144) [31] and JEG-3 (ATCC HTB-36) [32] cells were bought from ATCC.

### Testicular and cancer tissues for mircoarray analysis

Description of testicular tissue sample collection and preparation for cDNA microarray expression analysis was described in Biermann et al. [33]. Briefly, all testicular tissue samples were obtained immediately after orchiectomy. Normal testicular specimens were obtained from three patients with prostate carcinomas. All tumors were classified according to the WHO classification based on their histology as pT1 stage. Samples were examined by frozen section to assure a significant tumor cellularity. Immunohistochemical staining of CIS samples with an antibody to TFAP2C ensured that samples were composed of CIS only (100% of the tubules composed of CIS) without any normal testicular tubules or invasive tumors.

### DNA, RNA and protein isolation

DNA, RNA and proteins were isolated as described previously [34] [35]. Briefly, DNA was isolated by phenol-chloroform-isoamyl-alcohol extraction, RNA by TRIzol (Invitrogen, Karlsruhe, Germany) and proteins by RIPA buffer (50 mm TRIS-HCl pH 8, 150 mm NaCl, 1% NP-40, 0.5% sodium deoxycholate, 0.1% SDS +1 tablet of protease inhibitor cocktail (Roche, Mannheim, Germany)). DNA/RNA quality was assessed by photometric measurement of ratios 260/280 nm and 260/230 nm using a NanoDrop photometer (Peqlab, Erlangen, Germany).

### Western blot

Western blot analysis was performed as described previously [35]. See table 1 (Table S1) for antibody details and dilution ratios.

**Table 1:** Antibodies used in this study for IHC, western blotting and dot blotting.

Antibody	Company	Clone/Order No.	Origin	Western Blot	Dot Blot	IHC
5caC	Active Motif	61225	Rabbit	−	1:1500	1:1000
5fC	Active Motif	61223	Rabbit	−	1:1500	1:500
5hmC	Active Motif	39769	Rabbit	−	1:4000	1:1000
5mC	Abcam	33D3, ab10805	Mouse	−	1:5000	1:250
beta-Actin	Sigma-Aldrich	AC-15	Mouse	1:20000	1:20000	−
DNMT3B	Abcam	HPA001595	Mouse	−	−	1:200
OCT3/4	Santa Cruz	c-10	Mouse	1:500	−	−
TDG	Abcam	ab65219	Rabbit	1:500	−	−

### Immunohistochemistry

Tumor tissues were dissected, fixed in 4% phosphate-buffered formalin for 1–2 days at 4°C and processed in paraffin wax. 4 μm thick tissue sections on glass slides were pretreated in the Lab Vision PT Modul (Thermo Scientific, Munich, Germany) and in PT Modul Buffer (pH 6) (TA-250-PM, Medac, Hamburg, Germany) for 20 minutes and at 99°C, followed by a cool-down phase of 20 minutes at RT. Endogenous peroxidases were blocked by incubation in peroxidase blocking buffer (TA-125-HP, Medac, Hamburg, Germany) for 10 minutes. Primary antibodies were incubated for 30 minutes at RT. Signal detection was performed semiautomatically in the Autostainer 480 S (Medac, Hamburg, Germany) using the Bright Vision + polymer detection system (Medac, Hamburg, Germany) and the following settings: Enhancer for 10 minutes, polymer (Poly-HRP-Goat anti-mouse/-rabbit IgG) for 20 minutes, 3, 3′-diaminobenzidine (DAB) (415192F, Medac, Hamburg, Germany) for 8 minutes. Afterwards, nuclei were stained by hematoxylin for 3 minutes. See table 1 (Table S1) for antibody details and dilution ratios.

### Quantitative real-time RT-PCR

For first strand synthesis 500 ng of DNAse (NEB, Frankfurt, Germany) digested RNA template was used. First-strand cDNA synthesis was performed according to the RevertAid First Strand cDNA Synthesis Kit manual (Fermentas, St. Leon-Rot, Germany). For qRT-PCR 8,33 ng of each cDNA and the Maxima SYBR Green qPCR Master Mix (Fermentas, St. Leon-Rot, Germany) was used. PCR was performed at 94°C/30 s, 60°C/60 s for 40 cycles using the ViiA 7 Real Time PCR System (Applied Biosystems, distributed by Life Technologies, Carlsbad, CA, USA). At the end of each PCR run a melting point analysis was performed to confirm primer specificity. GAPDH was used as housekeeping gene and for data normalization. Each sample was analyzed in technical triplicates. See table 2 (Table S2) for primer sequences.

**Table 2:** Oligonucleotides used in this study for qRT-PCR analysis.

Gene	Forward primer	Reverse primer	Tan	Cycles
AID/AICDA	AGAGGCGTGACAGTGCTACA	TGTAGCGGAGGAAGAGCAAT	60°C	40
APOBEC1	CCCACTTGATTTCGTAGAGCA	CCAGAGACAGAGCACCATGA	60°C	40
DNMT1	ACCTGGCTAAAGTCAAATCC	ATTCACTTCCCGGTTGTAAG	60°C	40
DNMT3a	ACTACATCAGCAAGCGCAAG	CATCCACCAAGACACAATGC	60°C	40
DNMT3b	CCAGCTCTTACCTTACCATC	CAGACATAGCCTGTCGCTTG	56°C	40
DNMT3l	GCCGTACACAAGATCGAAGG	TTTGGGCTTTTTGGAAAGTG	60°C	40
GADD45a	TCAGCGCACGATCACTGTC	CCAGCAGGCACAACACCAC	60°C	40
GADD45b	GTCGGCCAAGTTGATGAAT	CACGATGTTGATGTCGTTGT	60°C	40
GAPDH	TGCCAAATATGATGACATCAAGAA	GGAGTGGGTGTCGCTGTTG	60°C	40
IDH1	GGCTGCTTGCATTAAAGGTT	TTTGGCCTGAGCTAGTTTGA	60°C	40
IDH2	TGAACTGCCAGATAATACGGG	CTGACAGCCCCCACCTC	60°C	40
MBD2	AACCCTGCTGTTTGGCTTAAC	CGTACTTGCTGTACTCGCTCTTC	60°C	40
MBD4	GGGCAAAAACCATTGTCAAG	TGATTTTCCCAAAGCCAGTC	60°C	40
SMUG1	CTCCTCCTCCAGGAAGCTCT	CTGCTGCAGTGCCTGTCAT	60°C	40
SOX17	GGCGCAGCAGAATCCAGA	CCACGACTTGCCCAGCAT	60°C	40
SOX2	CCCTGCTGAGAATAGGACAT	CCCTGCAGTACAACTCTATG	56°C	40
TDG	AGGAGCTTCAGCCATCAGTT	GAATGGAAGCGGAGAACG	60°C	40
TET1	GCTGCTGTCAGGGAAATCAT	ACCATCACAGCAGTTGGACA	60°C	40
TET2	CCAATAGGACATGATCCAGG	TCTGGATGAGCTCTCTCAGG	60°C	40
TET3	TCGGAGACACCCTCTACCAG	CTTGCAGCCGTTGAAGTACA	60°C	40

### Quantification of 5mC, 5hmC

The ELISA-based ‘MethylFlash Methylated DNA Quantification Kit (Colorimetric)’ and the ‘MethylFlash Hydroxymethylated DNA Quantification Kit (Colorimetric)’ (all from Epigentek, distributed by BioCat GmbH, Heidelberg, Germany) were used to quantify the amount of 5mC and 5hmC in the DNA of GCC cell lines. Quantification was performed in triplicates according to the manual. 100 ng of total DNA was used for 5mC quantification and 200 ng for 5hmC quantification. The initial incubation time was 90 min and the final developing time was 10 min. For absolute quantifications standard curves were generated by plotting the concentration of the positive control supplied with the assay against the optical density (OD) at 450 nm after performing the assay. For relative quantification the OD at 450 nm of 5 ng/µl of assay positive control was used as reference.

### DNA dot blot

Genomic DNA was spotted onto a positively charged nylon membrane (Roth, Karlsruhe, Germany). The DNA spots were air dried for 15 min and UV-crosslinked (20 sec, 1200J/cm^2^). Membranes were blocked in 5% dry milk powder in PBS/0,.1% Tween20 (PBSTM) for 1h at room temperature (RT). Afterwards, the membranes were washed in PBS for 3×5 min at RT. The membranes were incubated with the first antibody, diluted in 5% PBSTM for 3 h, followed by another wash step. The secondary antibody, also diluted in 5% PBSTM was applied for 1 h. After a final wash step, the membranes were incubated in 2 ml PierceSuper Signal West Pico chemiluminescent substrate (Perbio, Bonn, Germany) and the signal was detected using Kodak X-Ray films (Sigma-Aldrich, Taufkirchen, Germany). Staining of membranes by methylene blue (0,.04% methylene blue in 5M sodium actetate) confirmed equal loading. Swirling in distilled water destained membranes. See table 1 (Table S1) for antibody details and dilution ratios.

### Measuring TET/TDG/demethylating/DNMT1 activity/amount of nuclear extracts

Nuclear and cytoplasmic proteins were extracted from 1×10^7^ cells according to the manual of the ‘Nuclear Extract Kit’ (Active Motif, Carlsbad, CA, USA). The ELISA-based ‘Epigenase 5mC-Hydroxylase TET Activity/Inhibition Assay Kit (Colorimetric)’, ‘EpiQuik DNA Demethylase Activity/Inhibition Assay Kit’ and the ‘Epigenase TDG Activity/Inhibition Assay Kit (Colorimetric)’ (all from Epigentek, distributed by BioCat GmbH, Heidelberg, Germany) were used to measure TET, demethylating and TDG activity of GCC cell lines. ‘EpiQuik DNMT1 Assay Kit’ (Epigentek, distributed by BioCat GmbH, Heidelberg, Germany) was used to determine the amount of DNMT1 protein. Each measurement was performed in triplicates according to the manual. 5 μg of nuclear or cytoplasmic extract was used for each assay.

### Whole genome gene expression array analysis

The whole procedure has already been published [36]. The array was reanalyzed in context of this study.

### IDH1/IDH2 mutation analysis by pyrosequencing

Somatic sequence alterations of the codon R132 (IDH1; HGNC, Acc: 5382) and R172 (IDH2; HGNC, Acc: 5383) were investigated by pyrosequencing as recently described [20]. IDH1 PCR amplification primers flanking the R132 mutation hotspot within exon 4 of IDH1 were IDH1-fwd-5′-CACCATACGAAATATTCTGG-3′ and IDH1-rev-biotin-5′- CAACATGACTTACTTG ATCC-3′ that amplify 135 bp of genomic DNA. Single-stranded DNA templates were annealed to 0.4 mM sequencing primer IDH1-Py-5′-GTGAGTGGATGGGTAAAACC-3′ at RT. Similarly, an 86 bp fragment from exon 4 of IDH2 containing the R172 coding region was amplified using the primer set IDH2-fwd-5′-AAACATCCCACGC CTAGTCC-3′ and IDH2-rev-5′-biotin-CTCCACCCTGGCCTACCT-3′. IDH2 R172 was sequenced using the pyrosequencing primer IDH2-Py-5′-AGCCCATCACCATTG-3′. As positive control glioblastoma samples with confirmed IDH1/IDH2 mutations were included.

### Xenotransplantation of TCam-2 and 2102EP cells

Xeontransplantation was performed as described in a previously [37]. Briefly, TCam-2 and 2102EP cells were cultivated for eight days in standard growth medium. Cells were trypsinized, counted and 1×10^7^ cells were re-suspended in 500 µl of 4°C cold Matrigel (BD, Heidelberg, Germany) and injected into the flank of CD1 nude mice using a cold syringe.

### Statistical analysis

qRT-PCR analysis, quantification assays and activity measurements were performed in technical triplicates. Standard deviations were calculated and two-paired t-tests were performed to determine the significance of the data. Significance level α = 5%.

## Results

Since murine PGCs are able to actively demethylate their genome during their migration to the genital ridges [38], we asked if adult human gonocytes and germ cells of different developmental stages also display ADD.

First, we verified specificity of the 5mC and 5hmC antibodies by DNA dot blotting (Fig. S1). The 5mC antibody specifically bound a DNA oligonucleotide containing 5mC, but no 5hmC (positive control from Epigentek ‘MethylFlash Methylated DNA Quantification Kit (Colorimetric)’). Vice versa, the 5hmC antibody detected only a 5hmC containing oligonucleotide (positive control from Epigentek ‘MethylFlash Hydroxymethylated DNA Quantification Kit (Colorimetric)’).

**Figure 1:**
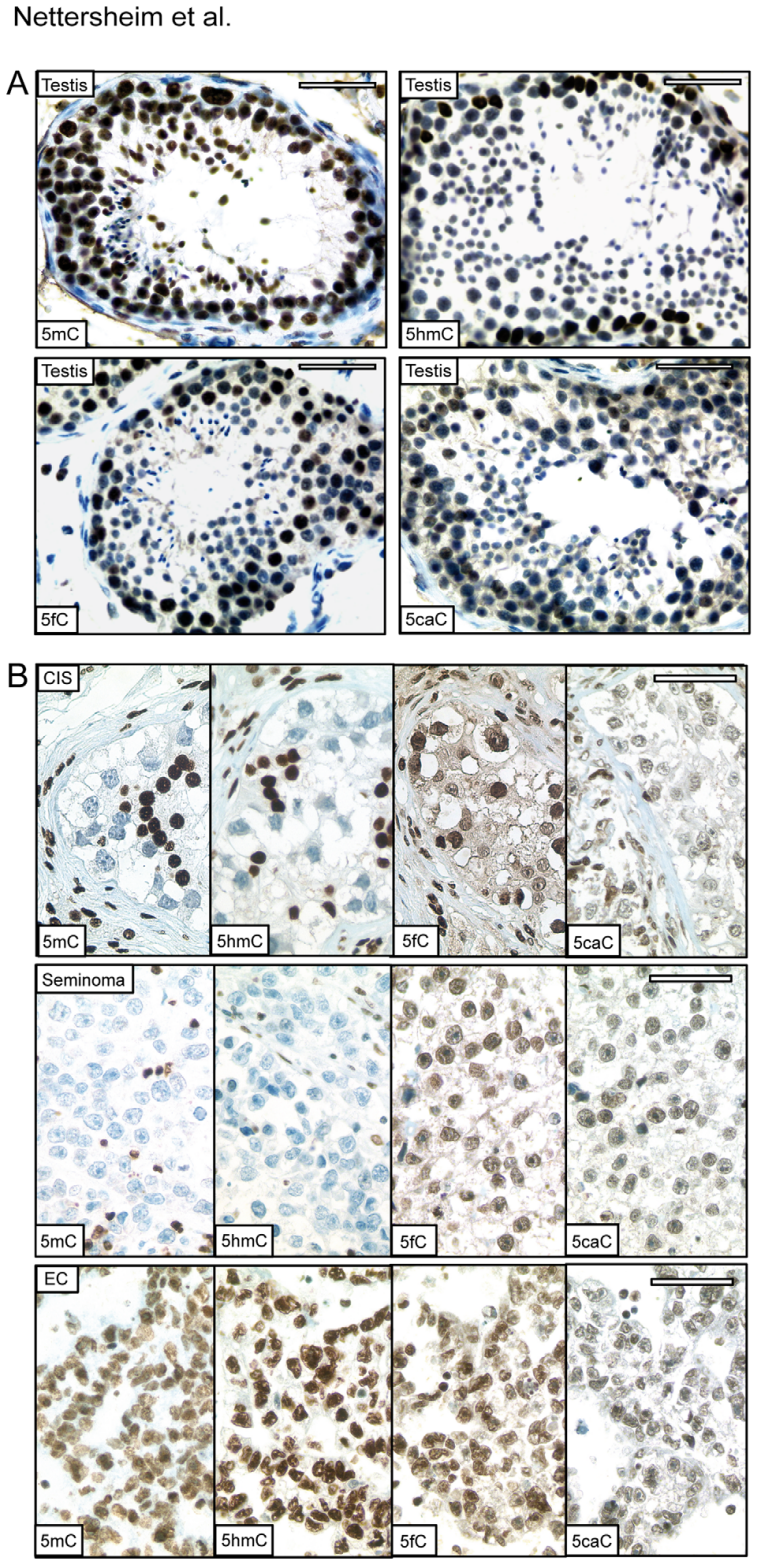
Immunohistochemical staining of cytosine modifications in testes sections and GCCs. A) IHC of 5mC, 5hmC, 5caC and 5fC in adult human healthy testis tissue and sperm (lower right corners). B) IHC of 5mC, 5hmC, 5fC and 5caC in CIS, seminoma and EC. Scale bars: 50 µm.

Next, we performed immunohistochemical staining (IHC) of adult human testis tissue sections detecting 5mC, 5hmC, 5fC and 5caC. We found a strong signal for 5mC from spermatogonia to post-meiotic spermatocytes. However, levels of 5hmC, 5fC and 5caC gradually decreased from the cells at the basal membrane to the cells located in the lumen of the tubules (Fig. 1A, Testis 5mC, 5hmC, 5fC, 5caC). The fact that all cell types of spermatogenesis display an equally strong signal for 5mC demonstrates that DNA methylation levels are maintained during spermatogenesis. However, the declining intensity of 5hmC, 5fC and 5caC staining during proceeding spermatogenesis suggests that either ADD is active in spermatogonia, but downregulated with onset of spermatogenesis or 5hmC, 5fC and 5caC are part of normal 5mC turnover in spermatogonia which is somehow blocked/reduced during spermatogenesis. This might lead to a gradual dilution of 5hmC, 5fC and 5caC during mitosis/meiosis, as already described by Inoue et al. during zygote development [39] [40].

Next, we extended our analysis to the precursor lesion CIS and various testicular GCC tissues (Fig. 1B). IHC demonstrated weak staining intensities for 5mC in the majority of CIS (97%, n = 72) and seminomas (71%, n = 211) (Fig. 1B, 5mC; Fig. S1). In contrast, embryonal carcinomas (96%, n = 74) (EC), teratomas (n = 5), yolk-sac tumors (n = 3) and choriocarcinomas (n = 4) show strong 5mC staining intensities (Fig. 1B, 5mC; Fig. S1). These findings confirm results of Netto et al. [41]. CIS (99%) and seminoma (78%) samples display also weak 5hmC staining intensities, while all other invasive GCC entities showed robust 5hmC levels (EC: 96%) (Fig. 1B, 5hmC; Fig. S1). Importantly, 5hmC (n = 22) could be detected in seminoma samples with moderate to strong 5mC staining intensities (n = 29) (Fig. S1). Interestingly, 5fC and 5caC could be detected in the vast majority (>90%) of all CIS, seminoma and EC tissues analysed (Fig. 1B; 5fC, 5caC).

**Figure 2:**
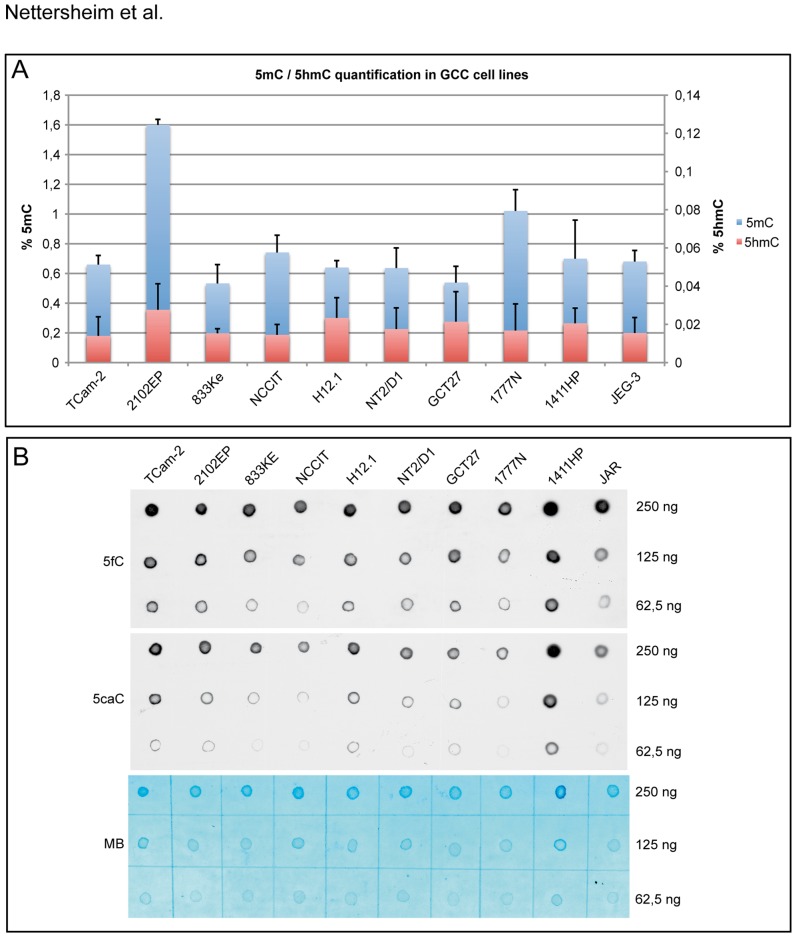
Analyses of cytosine modifications in GCC cell lines. A) Absolute quantification of 5mC and 5hmC levels in GCC cell lines (TCam-2 =  seminoma; 2102EP, 833K3, NCCIT, H12.1, NT2/D1, GCT27, 1777N, 1411HP  =  EC; JEG-3 =  choriocarcinoma). Levels were quantified in triplicates. Standard deviation is given at the top of each bar. B) DNA dot blot analysis of 5fC (5fC) and 5caC (5caC) in indicated GCC cell lines. 250–62.5 ng of total DNA was spotted on a positive-charged nylon membrane. A methylene blue staining (MB) was used as loading control.

ELISA-based absolute quantification of 5mC and 5hmC levels in DNA of GCC cell lines (1 seminoma, 8 EC and 1 choriocarcinoma cell line(s)) revealed that all cell lines display high levels of 5mC (0.53–1.60%), with the highest level in the EC cell line 2102EP (Fig. 2A), confirming the findings by Wermann et al. [42]. Li et al. analysed levels of 5hmC in different human tissues and cancer cell lines and demonstrated high levels of 5hmC in brain (0.67%), liver (0.46%), kidney (0.38%), colon (0.45%) and rectum (0.57%) tissue and little 5hmC content was detected in four cancer cell lines (Hela, HCT116, SW620, AN3Ca; <0.02%) [43]. Compared to these results, GCC cell lines also display low levels of 5hmC (0.014–0.027%). Of note, low levels of 5hmC had been demonstrated for several various cancer cell lines compared to normal, healthy tissues [44] [45]. Standard curves used for absolute quantification of 5mC and 5hmC levels are given in supplemental figures S2 (Fig. S2). A dot blot analysis demonstrated the presence of 5fC and 5caC in all GCC cell lines analyzed (Fig. 2 B, 5fC, 5caC). A methylene blue staining served as loading control (Fig. 2B, MB). IHC staining of global DNA methylation by a 5mC specific antibody further validated the 5mC status in three different GCC cell lines (Fig. S2), i.e. moderate 5mC levels in TCam-2, moderate to strong levels in NCCIT and high levels of 5mC in 2102EP. These results suggest that GCC cell lines are able to oxidize 5mC to 5hmC, 5fC and 5caC, which are part of the oxidation pathway of ADD.

**Figure 3:**
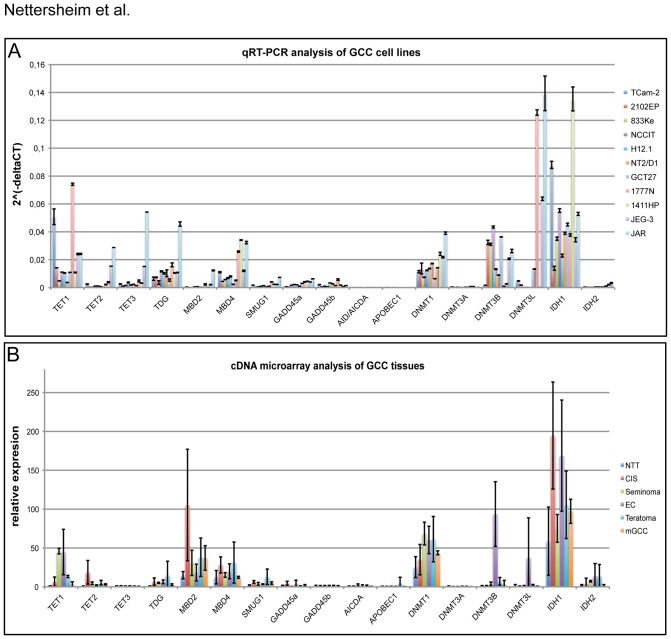
Meta-analysis of ADD keyplayer expression in GCC tissues and corresponding cell lines. A) qRT-PCR analysis of indicated genes in indicated GCC cell lines. GAPDH was used as a housekeeping gene and for data normalization. Standard deviation is indicated at the top of each bar. B) cDNA microarray analysis of indicated genes in normal testis tissue (NTT; n = 3), CIS (n = 3), seminoma (n = 4), embryonal carcinoma (EC; n = 3), teratoma (n = 3) and mixed germ cell cancers (mGCC; n = 3). Standard deviation is given at the top of each bar.

To analyze the expression of genes involved in ADD we performed quantitative real-time RT-PCR (qRT-PCR) analysis of the GCC cell lines and compared the results to data from a cDNA microarray of GCC tissues [46] (Fig. 3A, B). Seminoma and EC cell lines and tumors display most prominent expression of *TET1* while *TET2* and *TET3* are expressed at very low levels, suggesting that here *TET1* is the main molecule involved in 5mC oxidation. In the choriocarcinoma derived JAR cells, high expression of all *TETs* could be found, with the highest expression for *TET3* (Fig. 3A), suggesting that either TET3 is preferentially used for ADD or all three TET proteins can be utilized for ADD. We used an ELISA-based method to measure the demethylating activity of nuclear extracts and detected similar levels in the nuclear extracts of TCam-2, 2102EP and NCCIT cells, while demethylating activities of the cytoplasmatic extracts were comparably low (Fig. S3). Since *TET1* expression is much higher in TCam-2 than in 2102EP and NCCIT cells, but demethylation activities are comparable (Fig. 2A), we speculate that EC cells are also able to utilize different mechanisms to demethylate their genome. DNTM3B is highly expressed in ECs (Fig. 3A, B) and is assumed to be also a 5hmC dehydroxymethylase [47], so we assume that ECs are additionally able to utilize DNMT3B for ADD.

It has been demonstrated that mutations of the *IDH1 and IDH2* gene (*IDH1* ^R132^, *IDH2^R172^*) in glioblastomas are associated with the production of the TET inhibiting oncometabolite 2-hydroxyglutarate [17] [18] [19]. In the GCC cell lines analyzed here *IDH1*, but not *IDH2* is strongly expressed (Fig. 3A). We screened all analyzed GCC cell lines for mutations in the *IDH1* (*IDH*1^R132^) and *IDH2* (*IDH2* ^R172^) by pyrosequencing, but did not detect any mutations (Data S1, example given in Fig. S3). Glioblastoma samples with confirmed mutations of the *IDH1* or *IDH2* gene was used as positive controls. Therefore, it is unlikely that GCC cell lines accumulate the oncometabolite 2-hydroxyglutarate and reduce alpha-ketoglutarate levels that might influence TET enzyme mediated DNA demethylation.

The DNA glycosylases TDG, MBD4 and SMUG1 are thought to activate the BER at the end of the ADD cascade [8]. It is hypothesized that TDG is part of both, the deamination and oxidation pathway, while MBD4 and SMUG1 operate in the deamination pathway [8]. Since we hypothesized that the oxidation pathway of ADD is used in GCC cell lines, we believe that TDG is the main DNA glycosylase involved in this process. In line with this, we found a strong signal for TDG by western blotting (Fig. S3). Furthermore, we measured TDG activity by an ELISA-based principle and found comparably high TDG activity in the nuclear extract of TCam-2, 2102EP, NCCIT and JAR cells (Fig. S3).

The role of GADD45A/B, which is thought to recruit AID/AICDA and APOBECs during ADD is still subject to discussion [48] [49]. In GCC tissues and cell lines, *GADD45A*, *GADD45B*, *AID/AICDA* and *APOBEC1* are expressed at very low levels suggesting that these factors might not be essential for the ADD mechanism in GCC (Fig. 3A, B). These results indicate further that GCCs preferentially utilize the oxidation pathway of ADD.

Finally, we found comparable expression of the maintenance DNA methylase *DNMT1* on mRNA level in all cell lines and tissues, confirming the results by Netto et al. [41] who detected no differences in *DNMT1* expression between CIS/seminomas and non-seminomas. We verified this finding by measuring a similar DNMT1 protein amount in nuclear and cytoplasmatic extracts of seminomatous TCam-2 as well as EC cell lines 2102EP and NCCIT (Fig. S3). The de novo methylase *DNMT3A* is nearly absent in all GCC cell lines and tissues, while *DNMT3B* is highly expressed in most EC cell lines and tissues confirming previous results [50] [33]. Furthermore, the choriocarcinoma cell lines JEG-3 and JAR are strongly positive for *DNMT3B*, while the seminoma cell line TCam-2 and seminoma tissues displayed only very low expression levels, suggesting that seminomas posses only weak de novo DNA methylation activity, but are able to maintain DNA methylation patterns during replication by the maintenance DNA methyltransferase DNMT1. Strong expression of *DNMT3L* could be detected in both choriocarcinoma (JEG-3, JAR) and two EC (GCT27, 1777N) cell lines, while all other cell lines display absent or low expression of *DNMT3L*. These expression profile analysis suggests that all GCC tissues and cell lines utilize the oxidation pathway of ADD as well as replication-dependent maintenance DNA methylation (DNMT1), while de novo DNA methylation seems to be restricted to ECs and choriocarcinomas.

**Figure 4:**
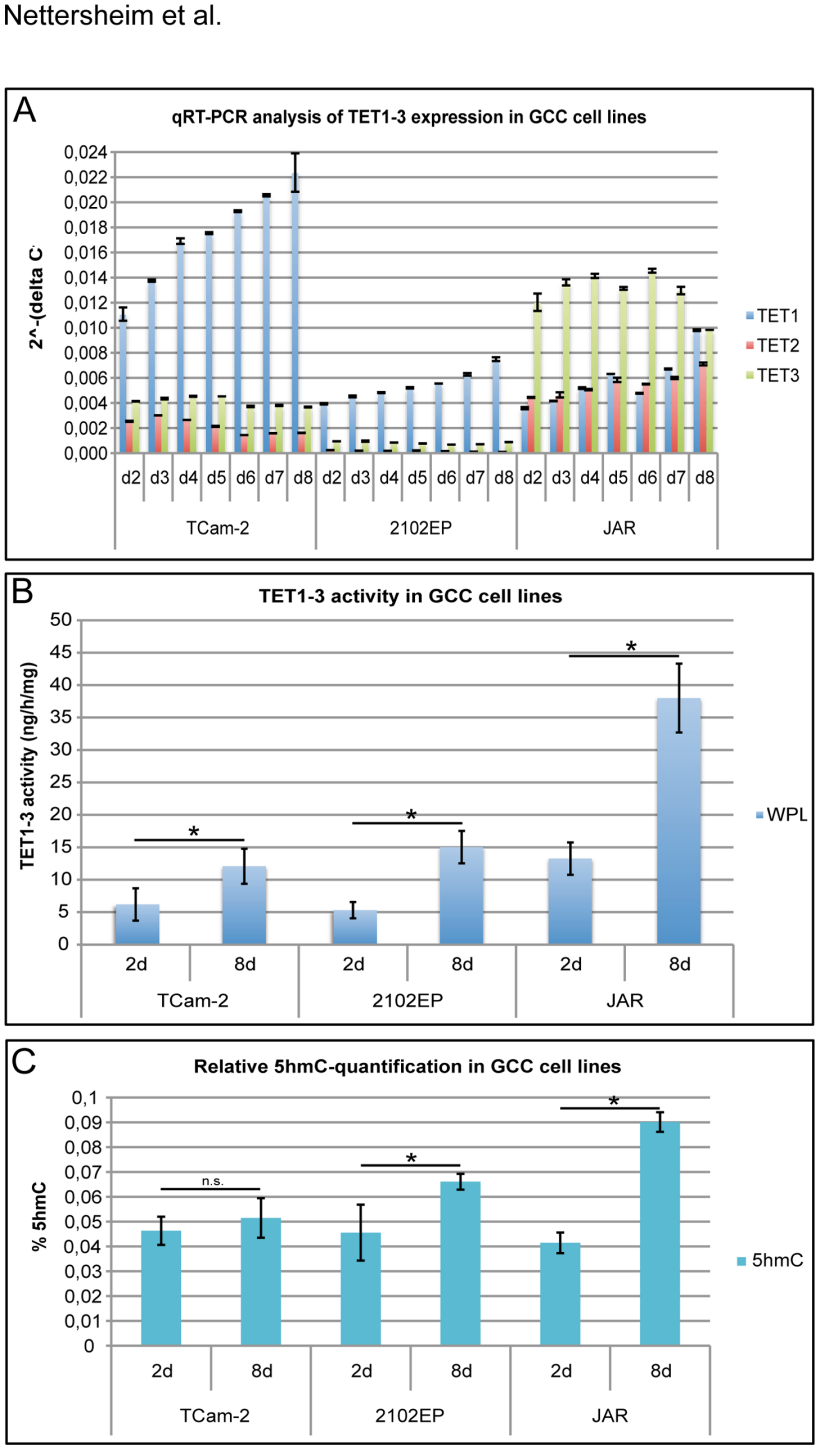
Analysis of TET expression/activity in three GCC cell lines. A) qRT-PCR analysis of TET1 -3 expression during 2–8 days of in vitro cultivation of TCam-2, 2102EP and JAR cells. Starting cell number: 1×10?4 cells. GAPDH was used as housekeeping gene and for data normalization. B) Measurement of total TET activity in whole protein lysate (WPL) of TCam-2, 2102EP and JAR cells at day 2 and 8 of in vitro cultivation. C) Relative quantification of 5hmC levels of TCam-2, 2102EP and JAR cells at day 2 and 8 of in vitro cultivation. Standard deviations are given at the top of each bar. Asterisks indicate a significant difference (p-value <0.05), while not significant changes are labelled as ‘n.s.’ (calculated by two-paired t-test).

During our experiments we recognized varying *TET1* mRNA levels in TCam-2 cells. To analyze this phenomenon in detail, we performed qRT-PCR analysis of *TET1*, *TET2* and *TET3* expression in TCam-2, 2102EP and JAR cells during 2–8 days of in vitro cultivation (Fig. 4A). We found that *TET1* expression levels increased over time in all cell lines analyzed (TCam-2: 2-fold; 2102EP: 2-fold; JAR, 2.7-fold), indicating that *TET1* expression correlates to cell density. In contrast, *TET2* expression decreased slightly over time in TCam-2, while in JAR cells *TET2* expression increased (1.6-fold). In TCam-2 and 2102EP cells, expression of *TET3* expression remained relatively constant. In JAR cells, *TET3* expression decreased after eight days. We measured the TET activity after 2 and 8 days of in vitro cultivation to correlate these data to the expression profile of the *TETs* (Fig. 4B). Here, the increased TET activity in TCam-2 and 2102EP cells is most likely due to the upregulation of *TET1* levels. In JAR cells, induction of TET activity was most pronounced and correlated to upregulation of *TET1* and *TET2*. Furthermore, in 2102EP and JAR cells the increase in *TET*/TET levels and activity clearly correlated with rising 5hmC levels (relative quantification to 5 ng/µl positive control provided in the kit; oligonucleotide containing either 50% 5mC or 20% 5hmC) (Fig. 4C). Of note, TCam-2 cells, despite displaying the highest levels of *TET1* when compared to 2102EP and JAR show only a very moderate TET activity. In addition, the increase in 5hmC levels was not significant (Fig. 4C).

**Figure 5:**
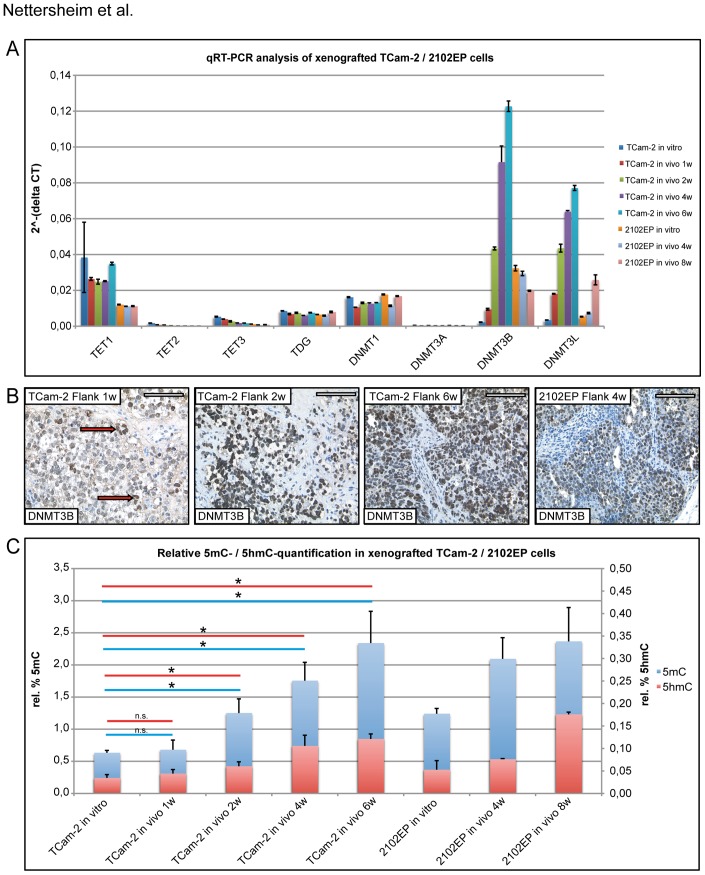
Analysis of ADD keyplayer expression and 5mC/5hmC levels after xenografting of TCam-2 and 2102EP cells. A) qRT-PCR analysis of indicated genes 1, 2, 4 and 6 weeks after xenografting of TCam-2 and 2102EP cells. GAPDH was used as housekeeping gene and for data normalization. Standard deviations are given at the top of each bar. B) IHC of DNMT3B 1, 2 and 6 weeks after xenografting of TCam-2 cells and 4 weeks after injection of 2102EP cells. Red arrows point at strongly DNMT3B positive cells. Scale bars: 100 µm. C) Relative quantification of 5mC and 5hmC levels of xenografted TCam-2 and 2102EP cells. Standard deviation is indicated above the bars. Significant changes are indicated as asterisks (p-value <0.05), while not significant changes are labelled as ‘n.s.’ (calculated by two-paired t-test).

Finally, we asked how expression of ADD keyplayers and cytosine modification levels (5mC/5hmC) are affected when seminomatous TCam-2 cells transit into an EC in the somatic microenvironment of the murine flank [37]. Therefore, TCam-2 cells were xenografted into the flank of nude mice; 2102EP cells served as an EC control. The marker genes *SOX17* and *SOX2* are differentially expressed between seminoma and ECs and serve as marker for transition [16]. Furthermore, TCam-2 cells downregulate *SOX17* and upregulate *SOX2* during in vivo transition to an EC [37]. Thus, we screened for changes in *SOX17* and *SOX2* expression as an indicator of a successful transition of TCam-2 cells to an EC-like tumor. qRT-PCR analysis demonstrates that *SOX17* expression gradually decreases, while *SOX2* is strongly upregulated during the time course of the xenograft experiment (Fig. S4). During the in vivo transition, we could not detect a significant change in *TET1* expression. *TET2* and *TET3* were slightly downregulated although overall expression levels were very low (Fig. 5A). Since expression of *TET1* is highly variable in TCam-2 cells, we determined the *TET1* expression levels at eighteen randomly picked time-points, ranging from 1*10∧4 cells to a confluent T75-flask and from one to eight days during cultivation of TCam-2. The average expression of *TET1* was calculated based on these values (Fig. S4). The high standard deviation reflects the variability of *TET1* expression in TCam-2 cells. Expression of *TDG* and *DNMT3A* (also very low) remained unchanged, while *DNMT1* expression slightly decreased (Fig. 5A). *DNMT3B* and *DNMT3L* were dramatically upregulated in tumors from xenografted TCam-2 cells, suggesting increased de novo DNA methylation (Fig. 5A). This increase was validated on protein level by IHC. The first cells that displayed a strong signal for DNMT3B were detected after one week (Fig. 5B, red arrows) and their number steadily and strongly increased. These results clearly demonstrate that upregulation of *DNMT3B* and its regulatory cofactor *DNMT3L* accompanies the transition of TCam-2 from seminoma-like to an EC-like tumor. Relative (to 5 ng/µl positive control provided in the kit; oligonucleotide containing either 50% 5mC or 20% 5hmC) quantification of 5mC and 5hmC levels demonstrates that both, 5mC and 5hmC levels increase (Fig. 5C), indicating that DNA de novo methylation and ADD accompanies the transition to an EC-like status.

## Discussion

In this study, we analyzed expression/activity of active and passive DNA (de)methylation keyplayers as well as presence of cytosine modifications (5mC/5hmC/5fC/5caC) during normal and malignant germ cell development. First, we could demonstrate that 5mC levels remain constant during spermatogenesis, while global 5hmC, 5caC and 5fC levels of the DNA declined. These data suggest that the oxidation pathway of ADD is active in spermatogonia but is turned off at later stages of spermatogenesis. Hence, levels and sites of 5mC modification become fixed reflecting a certain epigenetic ground state of sperm, with regard to methylation of imprinted and other epigenetically regulated loci [51] [52] [53]. In consequence this leads to conservation of this epigenetic ground state in sperm until fertilization of an oocyte.

We also analyzed expression of ADD keyplayers in GCC tissues and corresponding cell lines. We found the majority of CIS and seminoma tissues to be hypomethylated and hypohydroxymethylated compared to EC tissues. Therefore, we claim that induction of a process leading to DNA methylation and/or hydroxymethylation is a hallmark of non-seminomatous development from CIS. Interestingly, we detected 5fC and 5caC in all GCC tissues. With regard to CIS and seminomas, which present as weakly methylated and hydroxymethylated, the question arises when these 5fC and 5caC modifications were established.

Analysis of GCC cell lines showed that 5mC, 5hmC, 5fC, and 5caC are detectable in all samples, implicating that GCC cell lines utilize the oxidation pathway of ADD to demethylate their genome. Nevertheless, compared to regular somatic tissue the amounts of 5hmC in GCC cell lines measured are quite low, a fact already known for various other cancer cells and cancer derived cell lines [44] [43] [45].

Our study demonstrated comparable levels of 5mC, 5hmC and 5fC in the seminoma-like TCam-2 and in the non-seminomatous cell lines. This seminoma-atypical high methylation of TCam-2 cells has been described before [42]. Since our study reveals that 29% of seminomas are hypermethylated compared to CIS and the other seminoma samples analyzed (Fig. S1), it seems plausible that TCam-2 was derived from such a strongly methylated seminoma. Of note, it should be considered for future experiments that TCam-2 cells might not represent seminomas with low 5mC levels. Therefore, TCam-2 cells might behave different with respect to DNA methylation and treatment resistance compared to hypomethylated seminomas. Interestingly, Werman et al. showed that treatment of TCam-2 cells with the demethylating agent 5-aza-2′-deoxycytidine leads to an enhanced sensitivity towards cisplatin, demonstrating that the DNA methylation level of TCam-2 cells modulates drug resistance [42].

GCC cell lines and tissues display high levels of *TET1* expression, compared to *TET2* and *TET3* as well as moderate *TDG* expression and activity. In addition they show extremely low *GADD45A/B*, *AID/AICDA* and *APOBEC* expression, which were assumed essential for the deamination pathway [8]. It is unlikely that other *APOBEC* enzymes compensate for *APOBEC1*, since we detected only weak expression of other *APOBEC* family members in GCC tissues by cDNA microarray analysis (Fig. S4). The DNA glycosylase *MBD4* is expressed in GCC cell lines and tissues, but has been demonstrated to display a very low excision activity towards 5hmC, 5fC or 5caC [9]. So, we believe that MBD4 plays only a minor and subordinate role in ADD. In summary, the data suggest that GCC tissues and cell lines can utilize the oxidation pathway involving TET1 and TDG for ADD.

Seminomas and the seminoma-like cell line TCam-2 seem to utilize the replication dependent maintenance DNA methylation pathway since they display high *DNMT1* expression and low/absent *DNMT3A/B/L* levels. In non-seminoma tissues and cell lines, maintenance- and de novo- methylation seem to occur in parallel, indicated by expression of *DNMT1*, *DNMT3B* and *DNMT3L*. In line with this, comparable *DNMT1* expression levels between seminomas and non-seminomas and strong *DNMT3B* expression in ECs, but not seminomas have already been demonstrated [41] [33] [54]. In murine embryonic stem cells (ESC) de novo methylases *Dnmt3a/b* are highly expressed [55] [56] [57]. De novo methylase expression and 5mC levels are very low in PGCs [58] [2]. Seminomas are assumed to be the default developmental pathway of CIS cells, which are similar in gene expression and morphology to PGCs, while ECs are considered to be a malignant counterpart of ESCs, sharing a similar gene expression profile and differentiation potential. These similarities indicate that in general reduced 5mC levels and low de novo DNMT expression is a hallmark of PGC-like cells (PGC, CIS, seminoma), while ESC-like cells (ESC, EC) are hypermetyhlated compared to PGCs and possess de novo methyltransferase activity.

We could show that *TET1*, but not *TET2* or *TET3* expression increased with cell density in TCam-2 and 2102EP cells, while in JAR cells expression of both, *TET1* and *TET2* increased. Elevated *TET1* expression correlated with an increase in total TET activity and increase in 5hmC levels, demonstrating further that TET1 is the main oxygenase involved in oxidation of 5mC to 5hmC, 5fC and 5caC in these cell lines. JAR cells seem to be able to additionally utilize TET2 for 5mC oxidation.

A mutation in *IDH1* (*IDH1^R132^*) or *IDH2* (*IDH2^R172^*) causes the production of the TET inhibiting oncometabolite 2-hydroxyglutarate [44] [20]. Thus, despite TET proteins being present in GCCs, a block of 5mC oxidation might be a result of 2-hydroxyglutarate production. Sequencing revealed, that *IDH1*, which is highly expressed in GCC tissues and cell lines was not mutated in GCC cell lines, making an inhibition of the TETs by 2-hydroxyglutarate unlikely. *IDH2* expression is nearly absent in GCC cell lines and tissues and *IDH2* is not mutated (*IDH2^R172^*) in GCC cell lines. Low *IDH2* expression was also found in melanoma cells [59]. Additionally, Lian et al. could show that IDH2 plays an important role in maintaining proper 5hmC levels in melanocytes [59]. Thus, we hypothesize, that low levels of *IDH2* might contribute to overall low 5hmC levels in GCCs.

The transition of the seminomatous TCam-2 cells into an EC in the murine flank is accompanied by significantly increased *DNMT3B*, *DNMT3L* and 5mC levels, suggesting that de novo methylation is induced. In parallel, we measured an increase in 5hmC levels during the in vivo growth of TCam-2 cells. These findings indicate that both, de novo methylation and oxidation of 5mC to 5hmC are necessary to establish TCam-2s EC-like state in vivo. We hypothesize that genes important for a PGC-/CIS-/seminoma-like cell fate are silenced by de novo methylation, while EC associated genes become actively demethylated.

## Supporting Information

Figure S1 Analyses of cytosine modifications in GCC tissues and cell lines. A) Demonstration of specificity of 5mC and 5hmC antibody by DNA dot blotting. Indicated amounts (20–5 ng) of oligonucleotides containing either 5mC (50%) or 5hmC (20 %) were spotted on positive charged nylon membrane and detected either with 5mC or 5hmC antibody. B) Pie charts of 5mC/5hmC staining intensities in CIS, seminoma and EC tissues (in %). Staining intensities for 5mC were grouped in hypo- or hypermethylated, while 5hmC staining intensities were classified as hypo- or hyperhydroxymethylated. C) IHC of 5mC and 5hmC in teratoma, choriocarcinoma and yolk-sac tumor tissue. Secondary antibody controls are given in the lower right corner. Scale bar: 200 µm. D) IHC staining of 5mC, 5hmC, 5fC and 5caC in hypermethylated seminoma tissue. Scale bar: 100 µm.
(TIF)

Figure S2 Supporting information to 5mC/5hmC quantification. A, B) Standard curves used for absolute quantification of 5mC and 5hmC levels in GCC cell lines. C) IHC of 5mC in TCam-2, 2102EP and NCCIT cells. Secondary antibody controls are given in the lower right corners. Scale bars: 50 µm.
(TIF)

Figure S3 Supporting information to analysis of ADD mechanisms in GCC cell lines. A) Western blot analysis of OCT3/4 and TDG expression in nuclear (NE) and cytoplasmic (CE) extracts of TCam-2 (T), 2102EP (2) and NCCIT (N) cells. Membrane was stained by Coomassie blue. B) Measurement of demethylating activity of nuclear (NE)/cytoplasmic (CE) extracts of TCam-2, 2102EP and NCCIT. Standard deviations are given above each bar. Significant changes are labelled by an asterisk (p-value <0.05) (calculated by two-paired t-test). C) Western blot analysis of TDG expression in indicated GCC cell lines. b-actin served as housekeeper control. D) Measurement of TDG activity in the nuclear extracts (NE) of TCam-2, 2102EP, NCCIT and JAR cells. Standard deviations are given above each bar. E) Measurement of total DNMT1 protein amount in the nuclear (NE) and cytoplasmatic (CE) extracts of TCam-2, 2102EP and NCCIT cells. Standard deviations are given above each bar. Significant changes are labelled by an asterisk (p-value <0.05) (calculated by two-paired t-test).
(TIF)

Figure S4 Supporting information to xenograft experiments. A) qRT-PCR analysis of SOX2 and SOX17 expression at indicated time points in xenografted TCam-2 and 2102EP cells. GAPDH was used as housekeeping gene and for data normalization. Standard deviations are given at the top of each bar. B) qRT-PCR analysis of TET1 expression in eighteen different TCam-2 RNA isolates. Sample 19 represents the average expression. GAPDH was used as housekeeping gene and for data normalization. Standard deviation is given at the top of the last bar. C) cDNA microarray analysis of different APOBEC family members in normal testis tissue (NTT), CIS, seminoma, embryonal carcinoma (EC), teratoma and mixed germ cell cancers (mGCC). Standard deviation is given at the top of each bar.
(TIF)

Data S1 Results of the pyrosequencing-based mutation analysis of IDH1 (R132) and IDH2 (R172) in GCC cell lines.
(PDF)
