# Supplementary figures and images for: Analysis of TET Expression/Activity and 5mC Oxidation during Normal and Malignant Germ Cell Development
Source: PLoS One. 2013 Dec 26;8(12):e82881. doi: 10.1371/journal.pone.0082881 (PMC3873252; doi:10.1371/journal.pone.0082881)

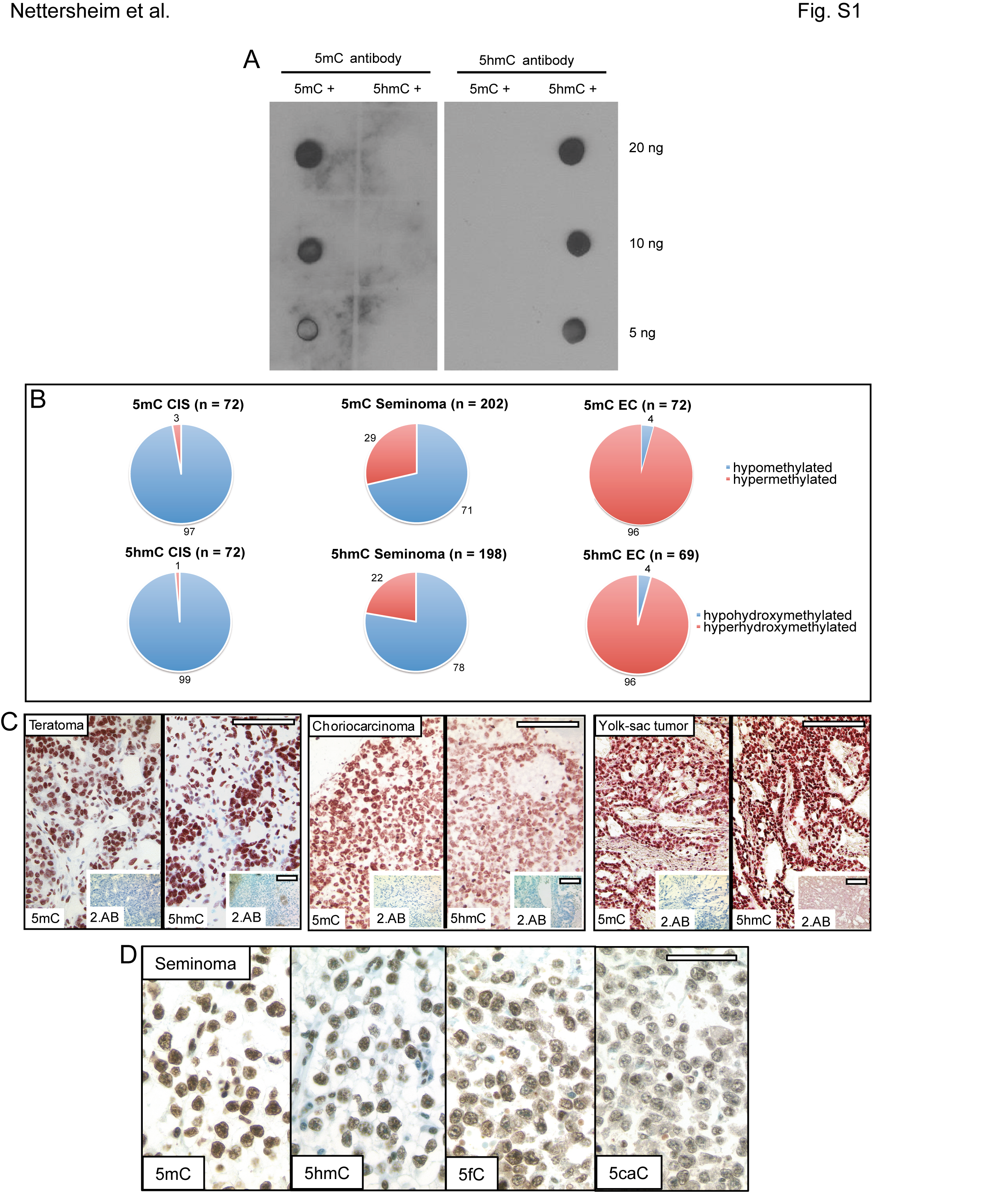

Supplement: Figure S1 — Analyses of cytosine modifications in GCC tissues and cell lines. A) Demonstration of specificity of 5mC and 5hmC antibody by DNA dot blotting. Indicated amounts (20–5 ng) of oligonucleotides containing either 5mC (50%) or 5hmC (20 %) were spotted on positive charged nylon membrane and detected either with 5mC or 5hmC antibody. B) Pie charts of 5mC/5hmC staining intensities in CIS, seminoma and EC tissues (in %). Staining intensities for 5mC were grouped in hypo- or hypermethylated, while 5hmC staining intensities were classified as hypo- or hyperhydroxymethylated. C) IHC of 5mC and 5hmC in teratoma, choriocarcinoma and yolk-sac tumor tissue. Secondary antibody controls are given in the lower right corner. Scale bar: 200 µm. D) IHC staining of 5mC, 5hmC, 5fC and 5caC in hypermethylated seminoma tissue. Scale bar: 100 µm. (TIF) [file pone.0082881.s001.tif]

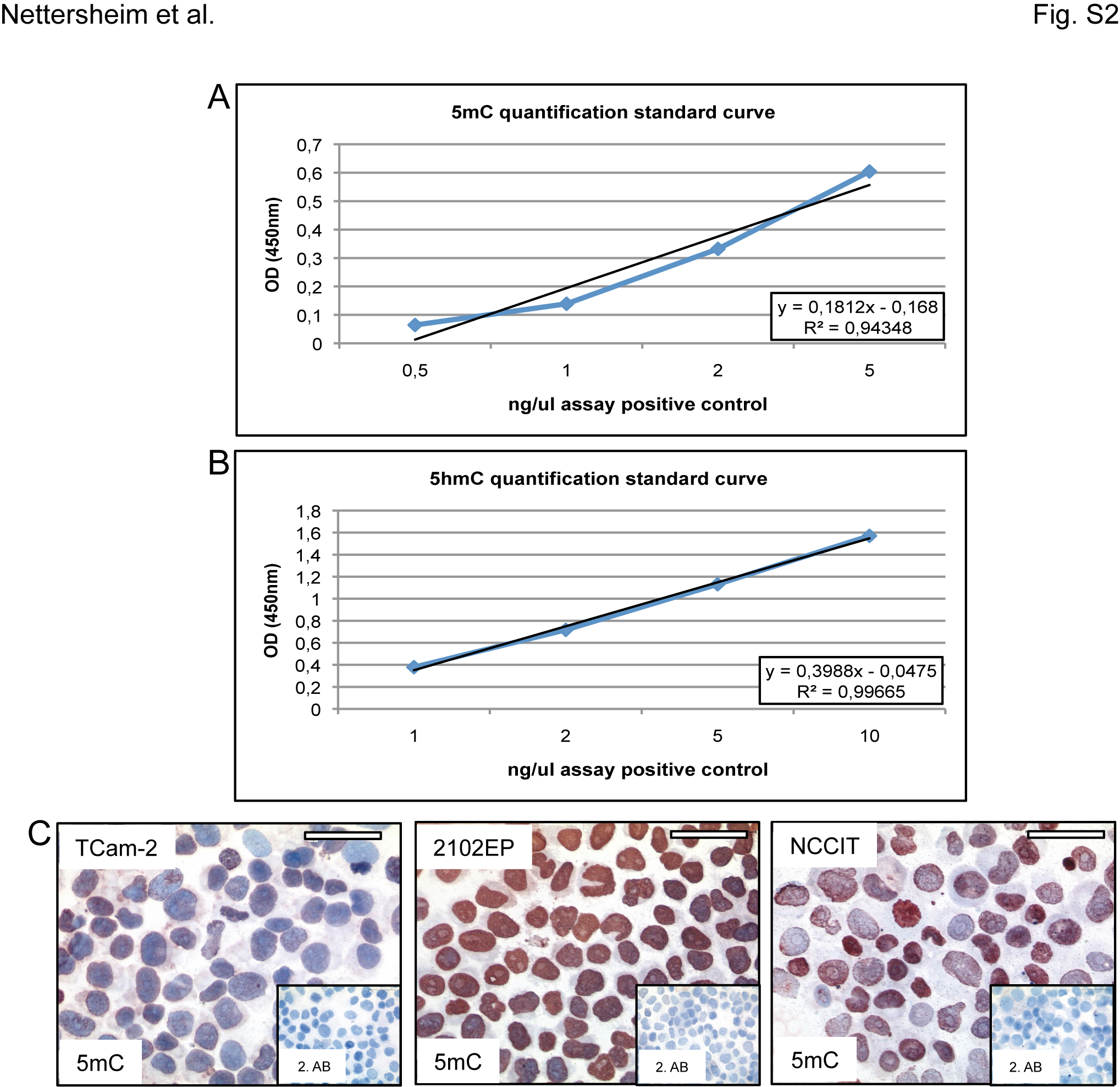

Supplement: Figure S2 — Supporting information to 5mC/5hmC quantification. A, B) Standard curves used for absolute quantification of 5mC and 5hmC levels in GCC cell lines. C) IHC of 5mC in TCam-2, 2102EP and NCCIT cells. Secondary antibody controls are given in the lower right corners. Scale bars: 50 µm. (TIF) [file pone.0082881.s002.tif]

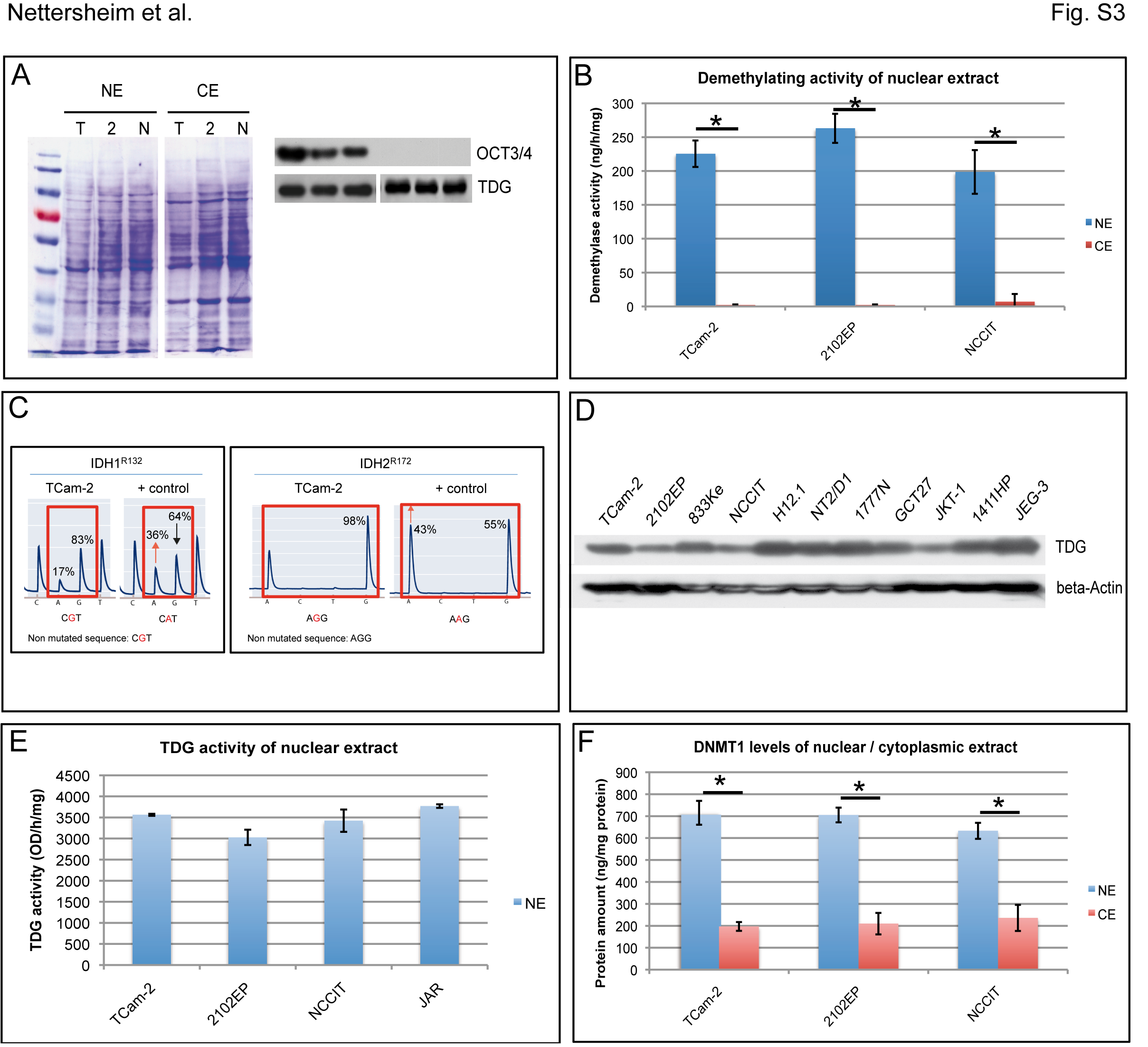

Supplement: Figure S3 — Supporting information to analysis of ADD mechanisms in GCC cell lines. A) Western blot analysis of OCT3/4 and TDG expression in nuclear (NE) and cytoplasmic (CE) extracts of TCam-2 (T), 2102EP (2) and NCCIT (N) cells. Membrane was stained by Coomassie blue. B) Measurement of demethylating activity of nuclear (NE)/cytoplasmic (CE) extracts of TCam-2, 2102EP and NCCIT. Standard deviations are given above each bar. Significant changes are labelled by an asterisk (p-value <0.05) (calculated by two-paired t-test). C) Western blot analysis of TDG expression in indicated GCC cell lines. b-actin served as housekeeper control. D) Measurement of TDG activity in the nuclear extracts (NE) of TCam-2, 2102EP, NCCIT and JAR cells. Standard deviations are given above each bar. E) Measurement of total DNMT1 protein amount in the nuclear (NE) and cytoplasmatic (CE) extracts of TCam-2, 2102EP and NCCIT cells. Standard deviations are given above each bar. Significant changes are labelled by an asterisk (p-value <0.05) (calculated by two-paired t-test). (TIF) [file pone.0082881.s003.tif]

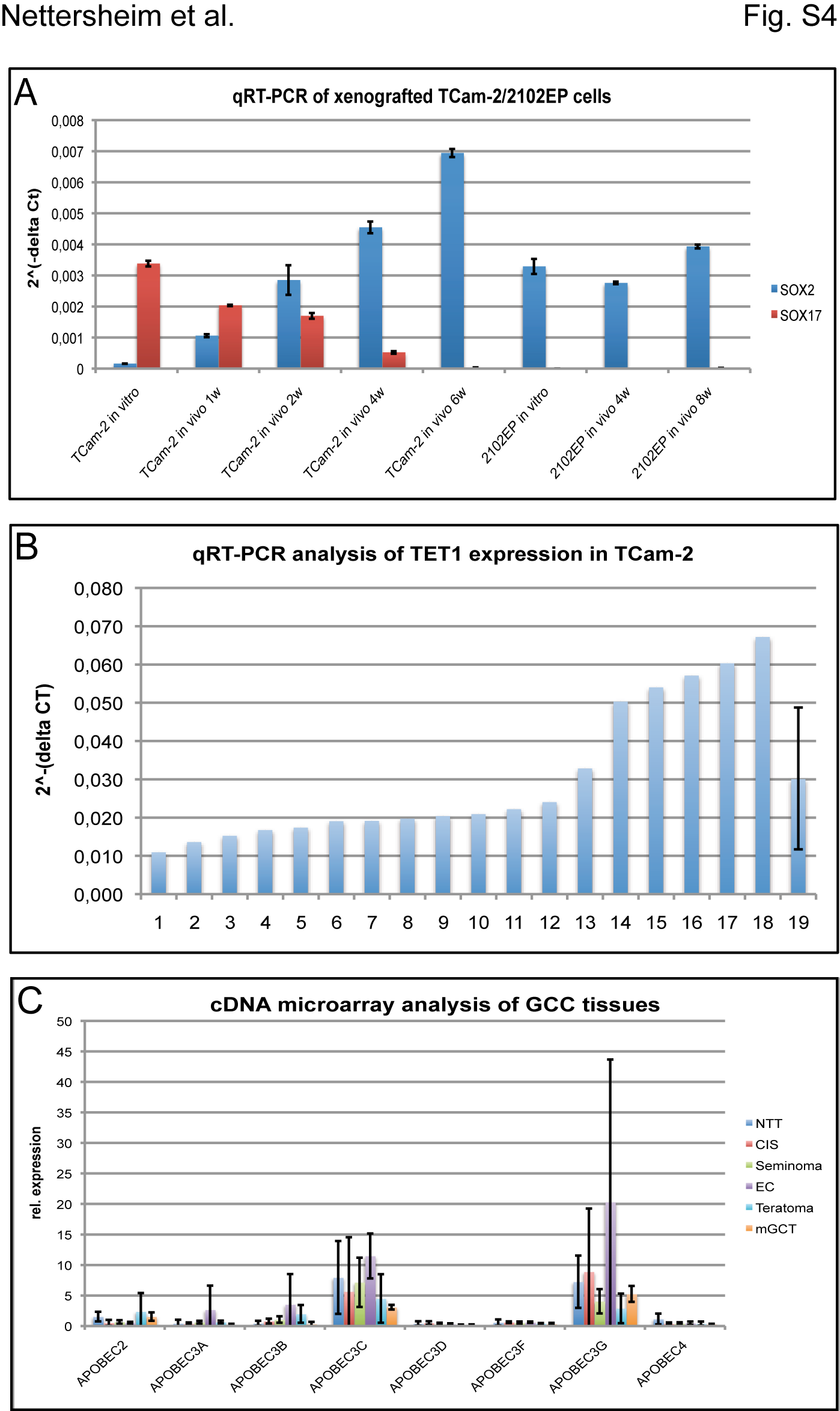

Supplement: Figure S4 — Supporting information to xenograft experiments. A) qRT-PCR analysis of SOX2 and SOX17 expression at indicated time points in xenografted TCam-2 and 2102EP cells. GAPDH was used as housekeeping gene and for data normalization. Standard deviations are given at the top of each bar. B) qRT-PCR analysis of TET1 expression in eighteen different TCam-2 RNA isolates. Sample 19 represents the average expression. GAPDH was used as housekeeping gene and for data normalization. Standard deviation is given at the top of the last bar. C) cDNA microarray analysis of different APOBEC family members in normal testis tissue (NTT), CIS, seminoma, embryonal carcinoma (EC), teratoma and mixed germ cell cancers (mGCC). Standard deviation is given at the top of each bar. (TIF) [file pone.0082881.s004.tif]
