# Supplementary material for: Analysis of TET Expression/Activity and 5mC Oxidation during Normal and Malignant Germ Cell Development
Source: PLoS One. 2013 Dec 26;8(12):e82881. doi: 10.1371/journal.pone.0082881 (PMC3873252; doi:10.1371/journal.pone.0082881)
Supplement: Data S1 — Results of the pyrosequencing-based mutation analysis of IDH1 (R132) and IDH2 (R172) in GCC cell lines. (PDF) [file pone.0082881.s005.pdf]

AQ Full Report

Run Info

Run Name	IDH1 25.06.12
Operator	NP-2342F08B15F7\Andreas Waha
Run Date/Time	25.06.2012 11:25:38
Instrument Name	PyroMark Q24
Serial Number	000019
Instrument Method	PyroMark Q24 Method 001 Rev. A
Plate ID	
Barcode	
Reagent ID	
Run Note	

Run Log

0h 0min 0s	information	Run started

Analysis results

Well: A1
Assay: IDH1-1
Sample ID: JAR
Note:
Analysis version: 1.0.10

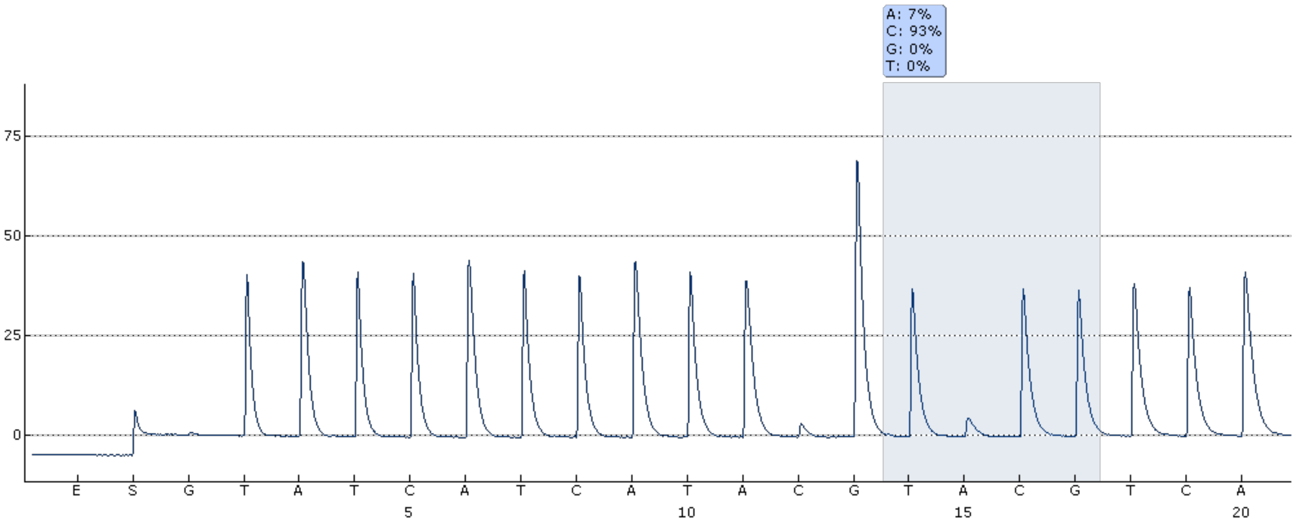

Sequence to analyze:
TATCATCATAGGTNGTCATGC

Position	1
Quality	Passed
A (%)	7
C (%)	93
G (%)	0
T (%)	0

No warnings.

Well: A2
Assay: IDH1-1
Sample ID: JKT-1
Note:
Analysis version: 1.0.10

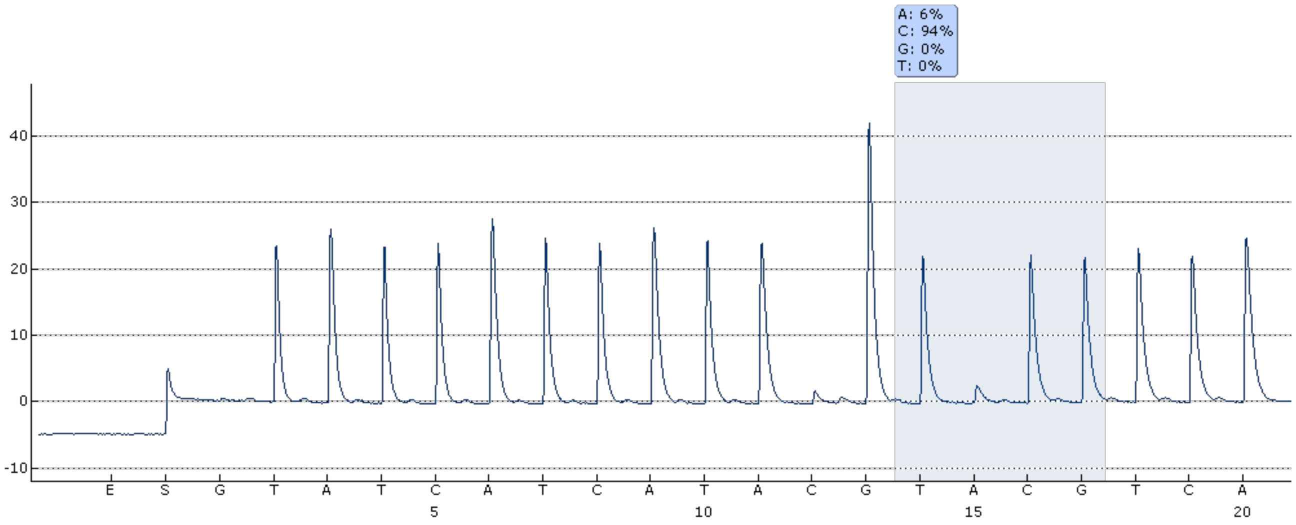

Sequence to analyze:
TATCATCATAGGTNGTCATGC

Position	1
Quality	Passed
A (%)	6
C (%)	94
G (%)	0
T (%)	0

No warnings.

Well: A3
Assay: IDH1-1
Sample ID: NCCIT
Note:
Analysis version: 1.0.10

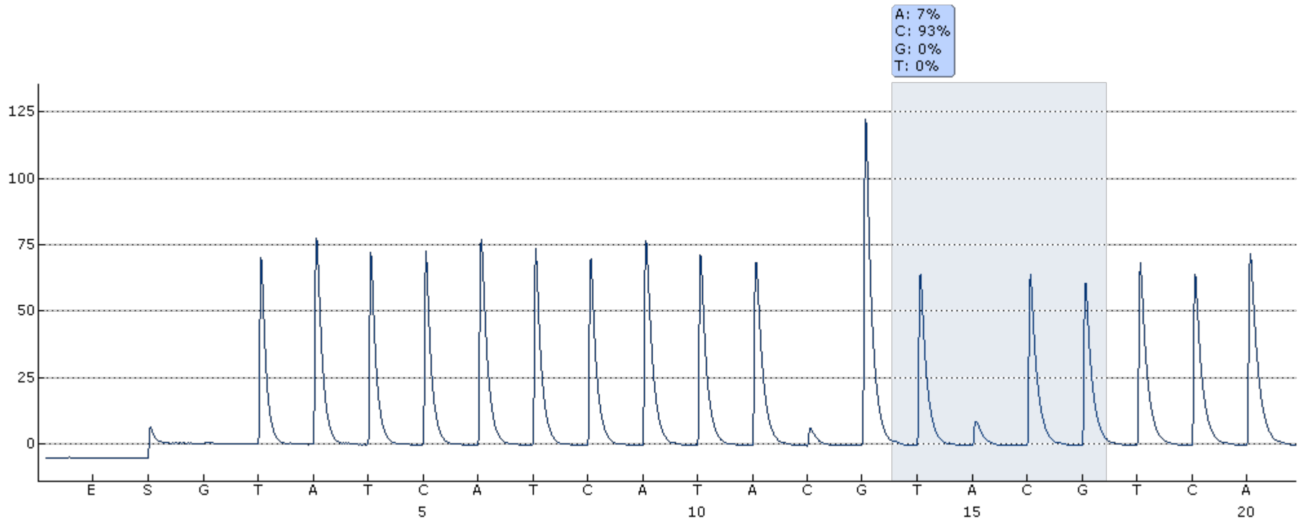

Sequence to analyze:
TATCATCATAGGTNGTCATGC

Position	1
Quality	Passed
A (%)	7
C (%)	93
G (%)	0
T (%)	0

No warnings.

Well: A4
Assay: IDH1-1
Sample ID: TCam-2
Note:
Analysis version: 1.0.10

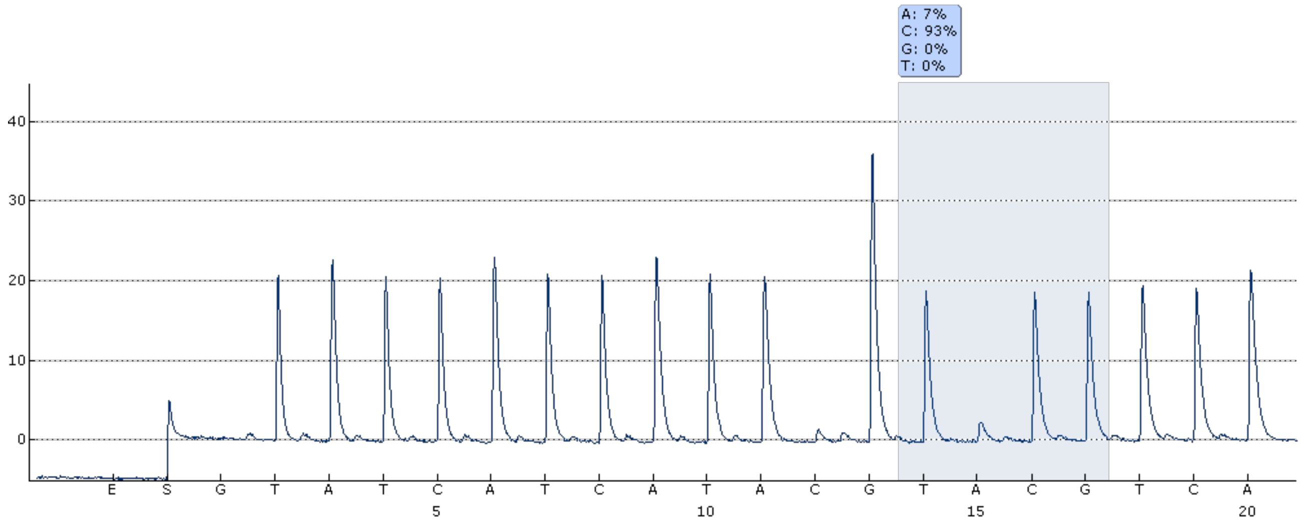

Sequence to analyze:
TATCATCATAGGTNGTCATGC

Position	1
Quality	Passed
A (%)	7
C (%)	93
G (%)	0
T (%)	0

No warnings.

Well: A5
Assay: IDH1-1
Sample ID: NT2
Note:
Analysis version: 1.0.10

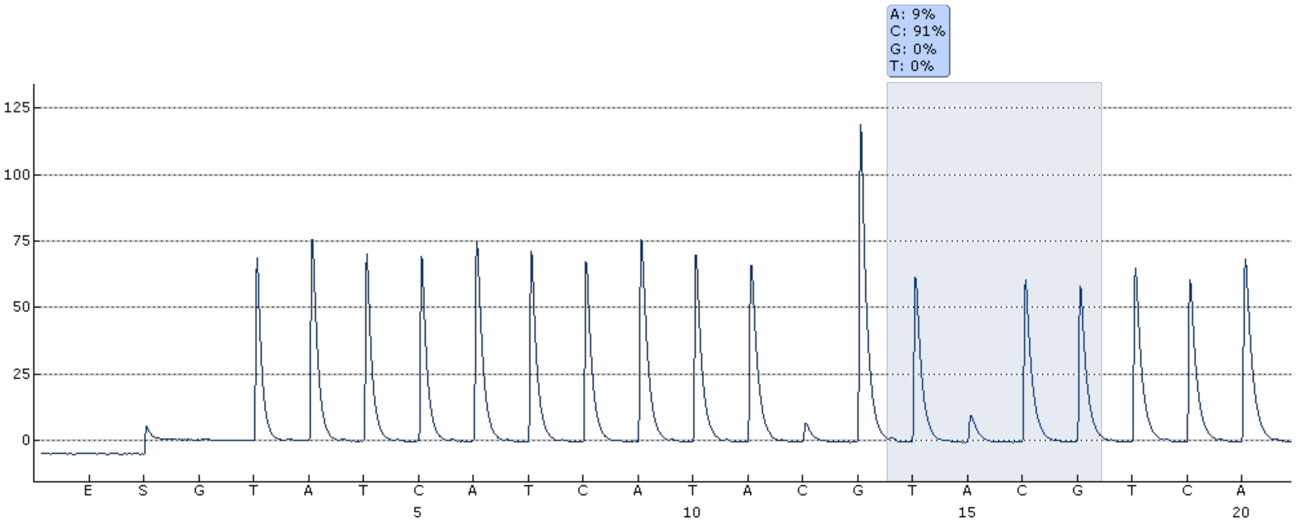

Sequence to analyze:
TATCATCATAGGTNGTCATGC

Position	1
Quality	Passed
A (%)	9
C (%)	91
G (%)	0
T (%)	0

No warnings.

Well: A6
Assay: IDH1-1
Sample ID: JEG-3
Note:
Analysis version: 1.0.10

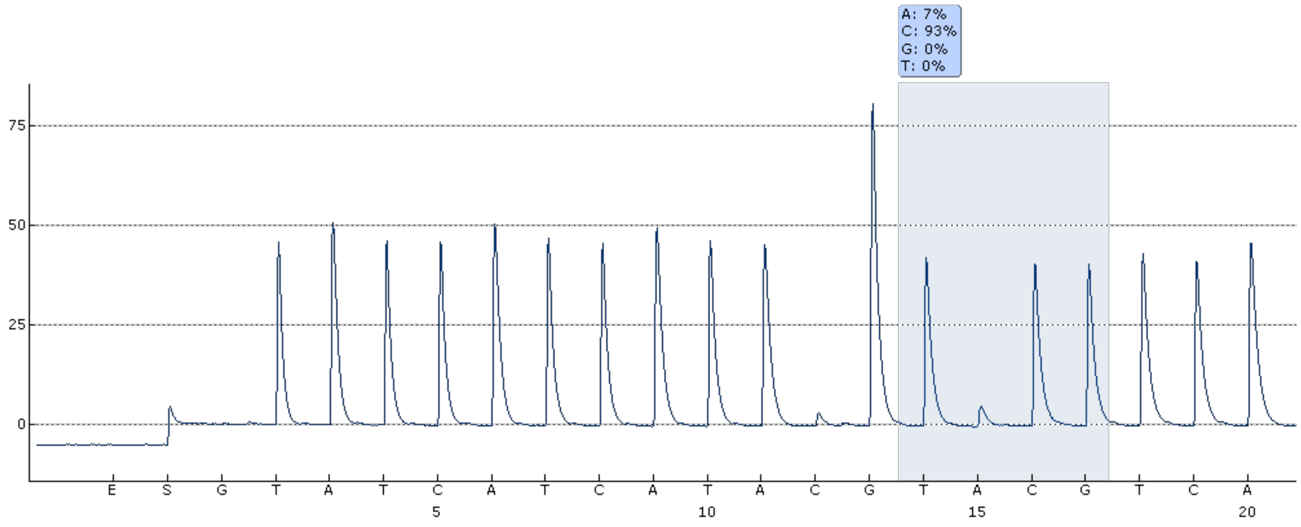

Sequence to analyze:
TATCATCATAGGTNGTCATGC

Position	1
Quality	Passed
A (%)	7
C (%)	93
G (%)	0
T (%)	0

No warnings.

Well: A7
Assay: IDH1-1
Sample ID: CCT
Note:
Analysis version: 1.0.10

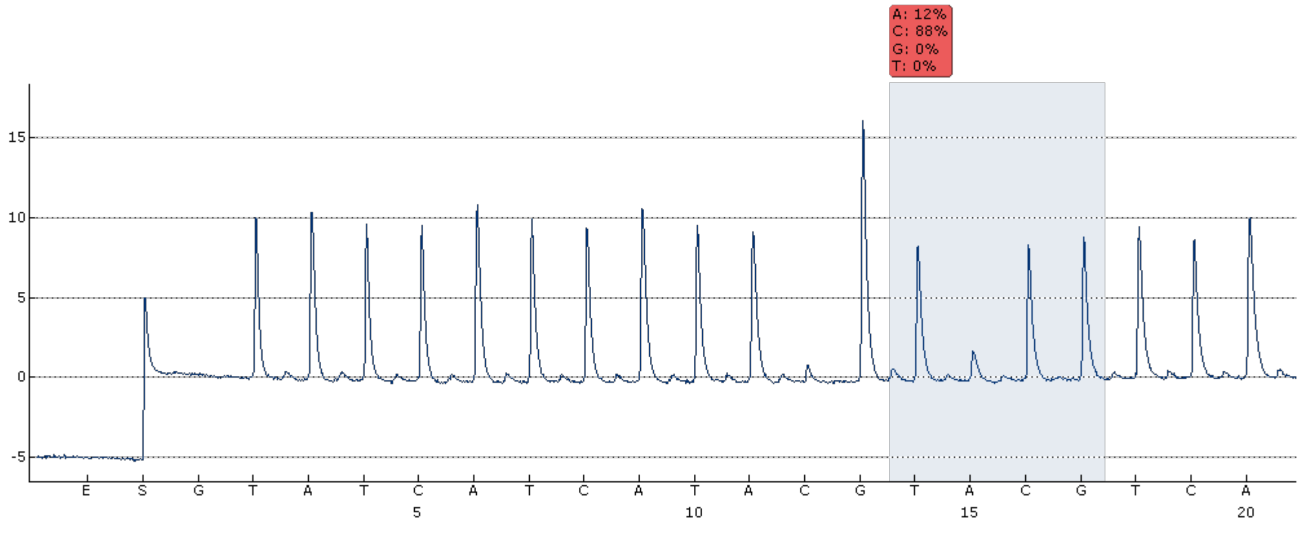

Sequence to analyze:
TATCATCATAGGTNGTCATGC

Position	1
Quality	Failed
A (%)	12
C (%)	88
G (%)	0
T (%)	0

Warnings:
Position 1: Failed due to low peak height.

Well: A8
Assay: IDH1-1
Sample ID: 2102 EP
Note:
Analysis version: 1.0.10

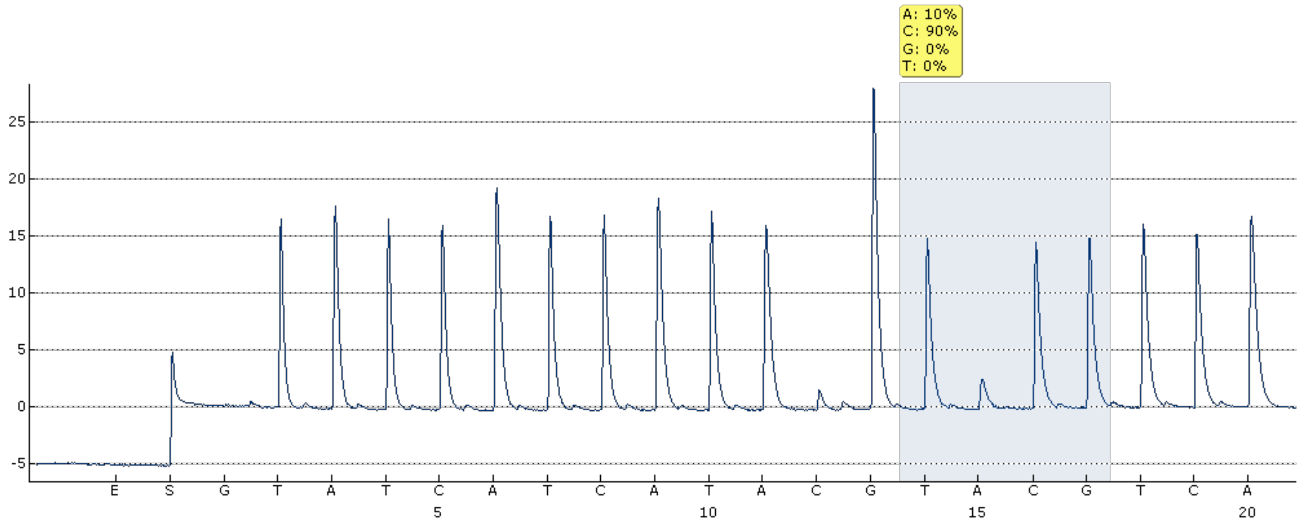

Sequence to analyze:
TATCATCATAGGTNGTCATGC

Position	1
Quality	Check
A (%)	10
C (%)	90
G (%)	0
T (%)	0

Warnings:
Position 1: Uncertain due to low peak height.

Well: B1
Assay: IDH1-1
Sample ID: H12
Note:
Analysis version: 1.0.10

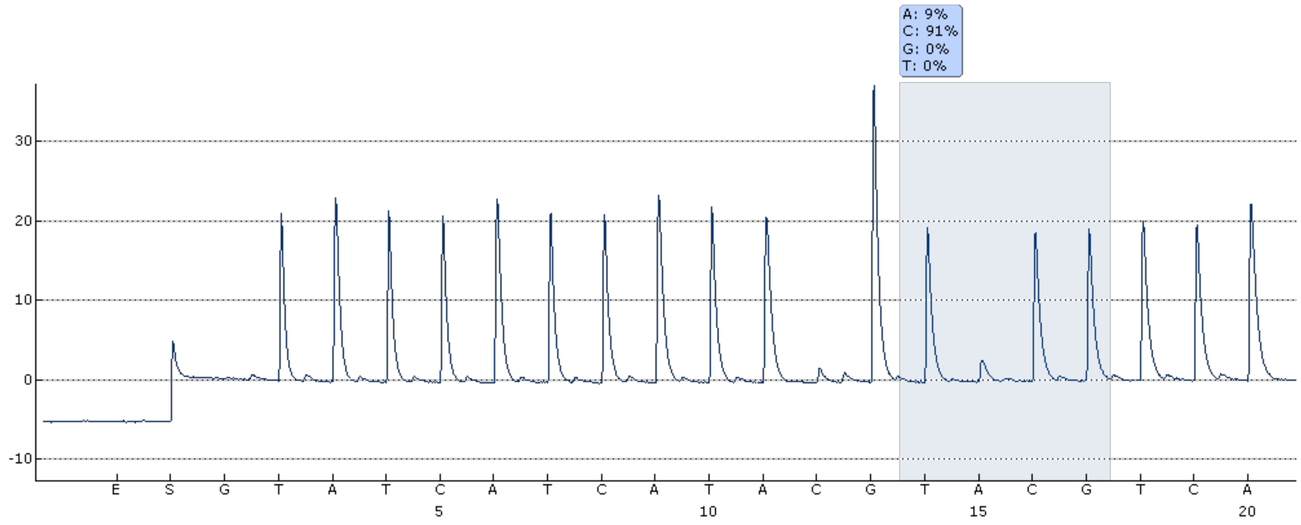

Sequence to analyze:
TATCATCATAGGTNGTCATGC

Position	1
Quality	Passed
A (%)	9
C (%)	91
G (%)	0
T (%)	0

No warnings.

Well: B2
Assay: IDH1-1
Sample ID: 577 M
Note:
Analysis version: 1.0.10

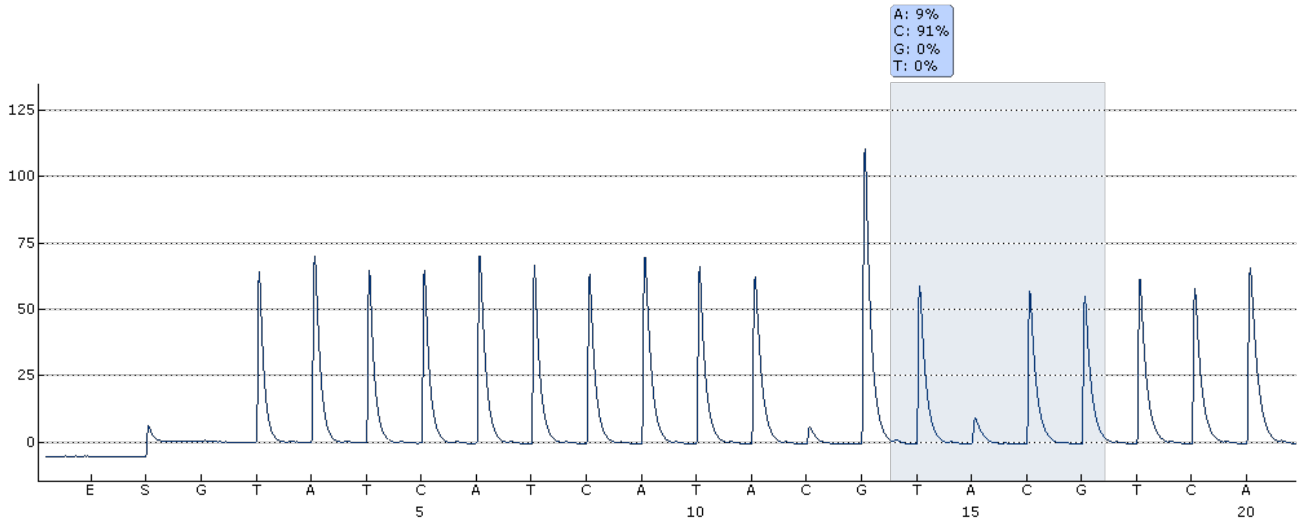

Sequence to analyze:
TATCATCATAGGTNGTCATGC

Position	1
Quality	Passed
A (%)	9
C (%)	91
G (%)	0
T (%)	0

No warnings.

Well: B3
Assay: IDH1-1
Sample ID: 833 K
Note:
Analysis version: 1.0.10

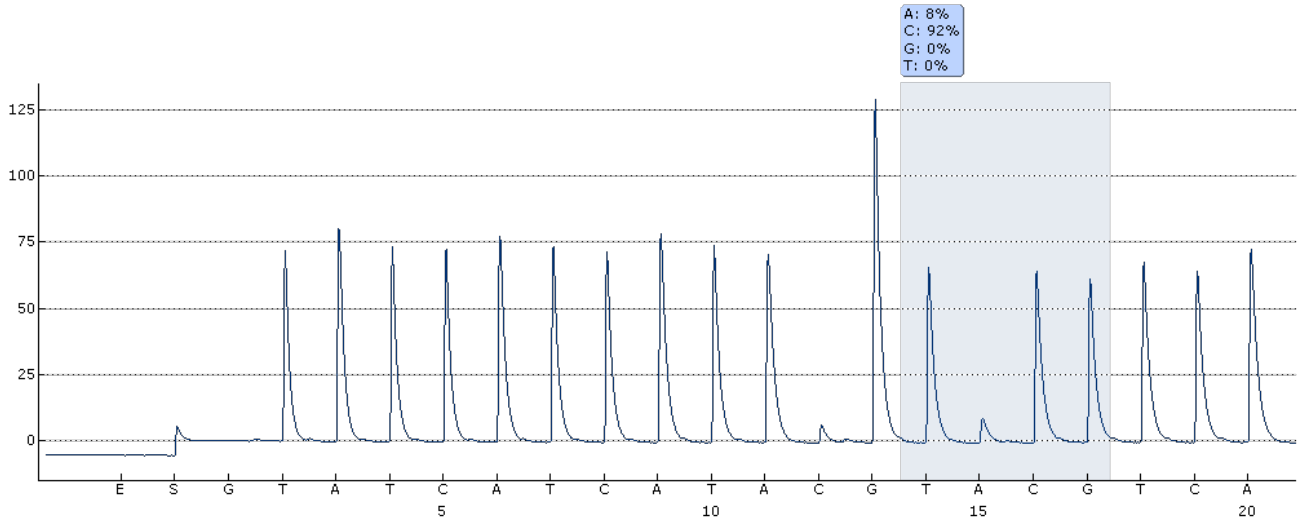

Sequence to analyze:
TATCATCATAGGTNGTCATGC

Position	1
Quality	Passed
A (%)	8
C (%)	92
G (%)	0
T (%)	0

No warnings.

Well: B4
Assay: IDH1-1
Sample ID: 1411
Note:
Analysis version: 1.0.10

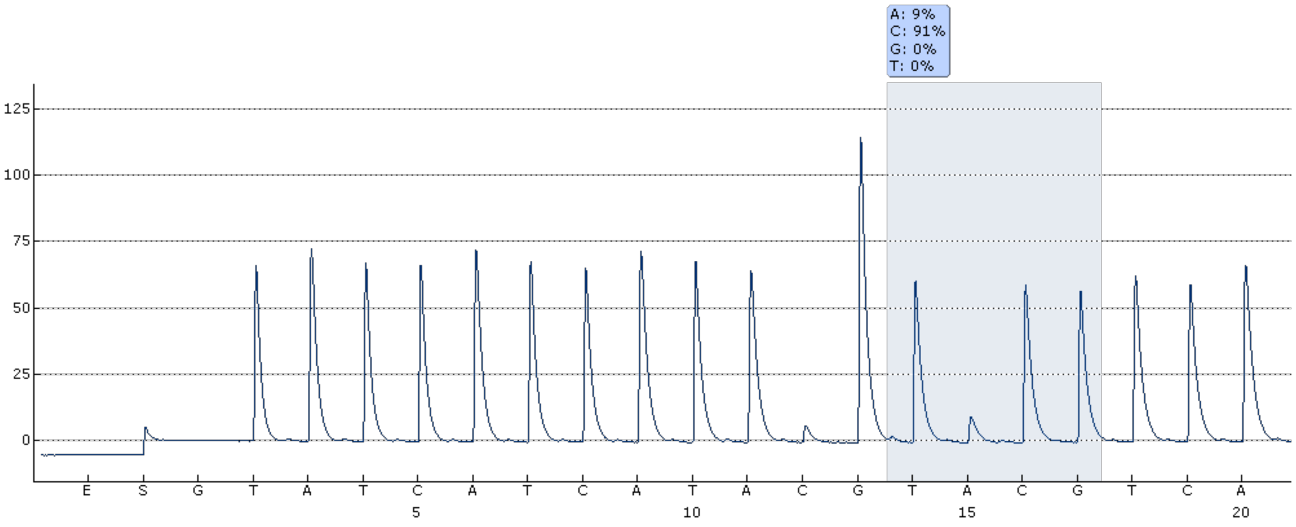

Sequence to analyze:
TATCATCATAGGTNGTCATGC

Position	1
Quality	Passed
A (%)	9
C (%)	91
G (%)	0
T (%)	0

No warnings.

Well: B5
Assay: IDH1-2
Sample ID: JAR
Note:
Analysis version: 1.0.10

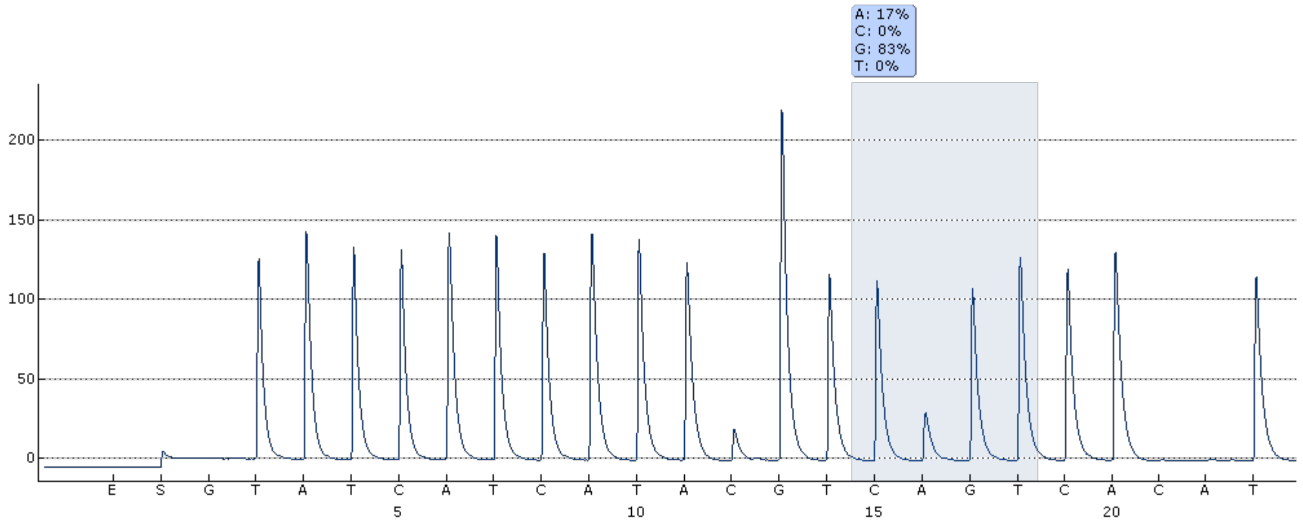

Sequence to analyze:
TATCATCATAGGTCNTCATGCTTAT

Position	1
Quality	Passed
A (%)	17
C (%)	0
G (%)	83
T (%)	0

No warnings.

Well: B6
Assay: IDH1-2
Sample ID: JKT-1
Note:
Analysis version: 1.0.10

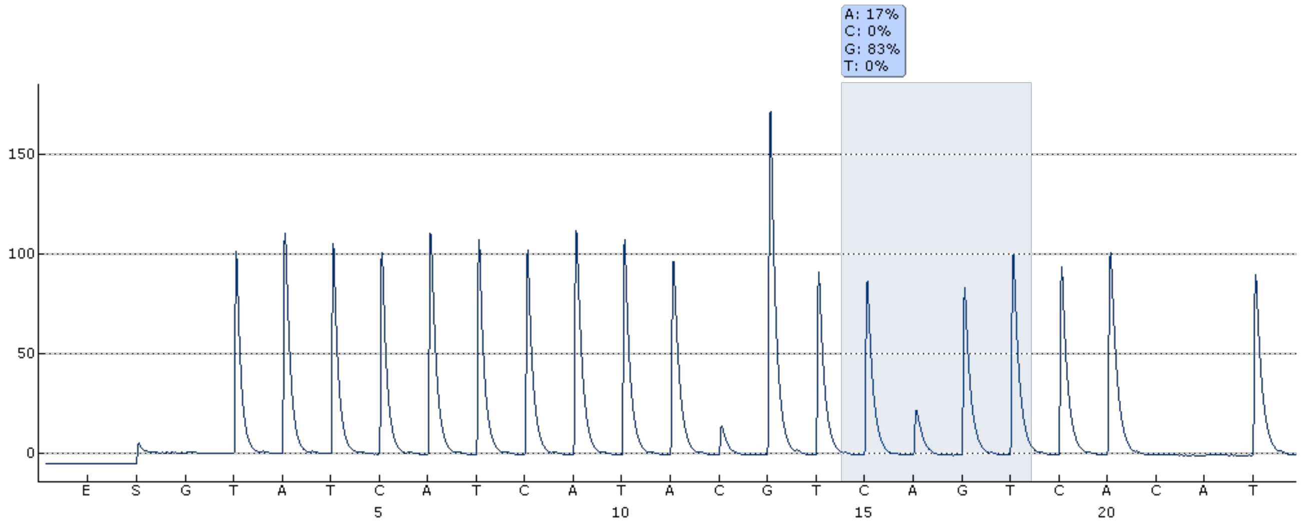

Sequence to analyze:
TATCATCATAGGTCNTCATGCTTAT

Position	1
Quality	Passed
A (%)	17
C (%)	0
G (%)	83
T (%)	0

No warnings.

Well: B7
Assay: IDH1-2
Sample ID: NCCIT
Note:
Analysis version: 1.0.10

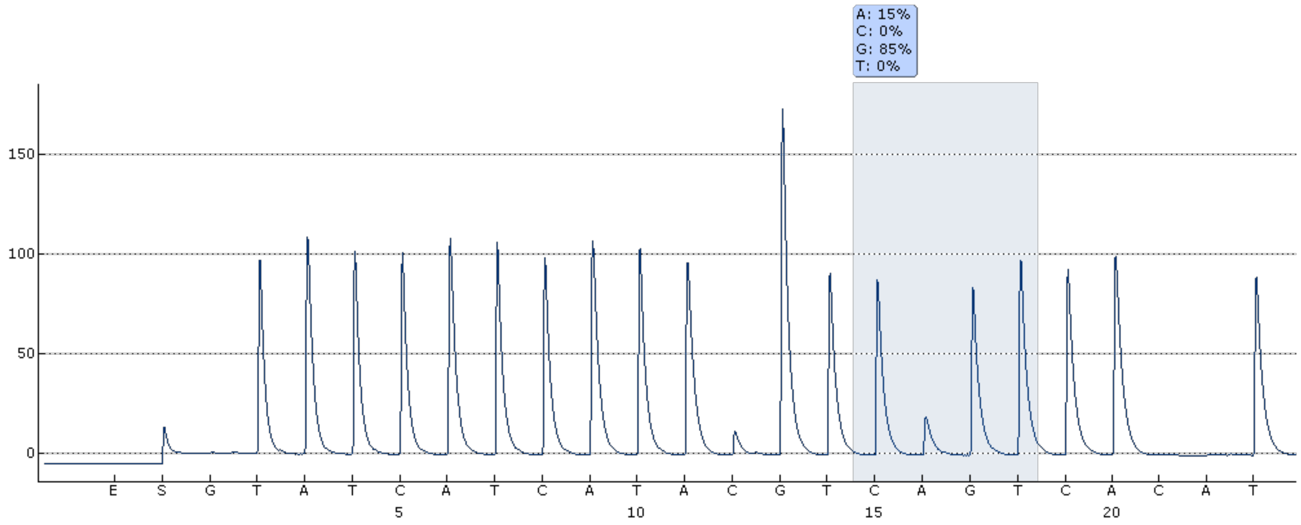

Sequence to analyze:
TATCATCATAGGTCNTCATGCTTAT

Position	1
Quality	Passed
A (%)	15
C (%)	0
G (%)	85
T (%)	0

No warnings.

Well: B8
Assay: IDH1-2
Sample ID: TCam-2
Note:
Analysis version: 1.0.10

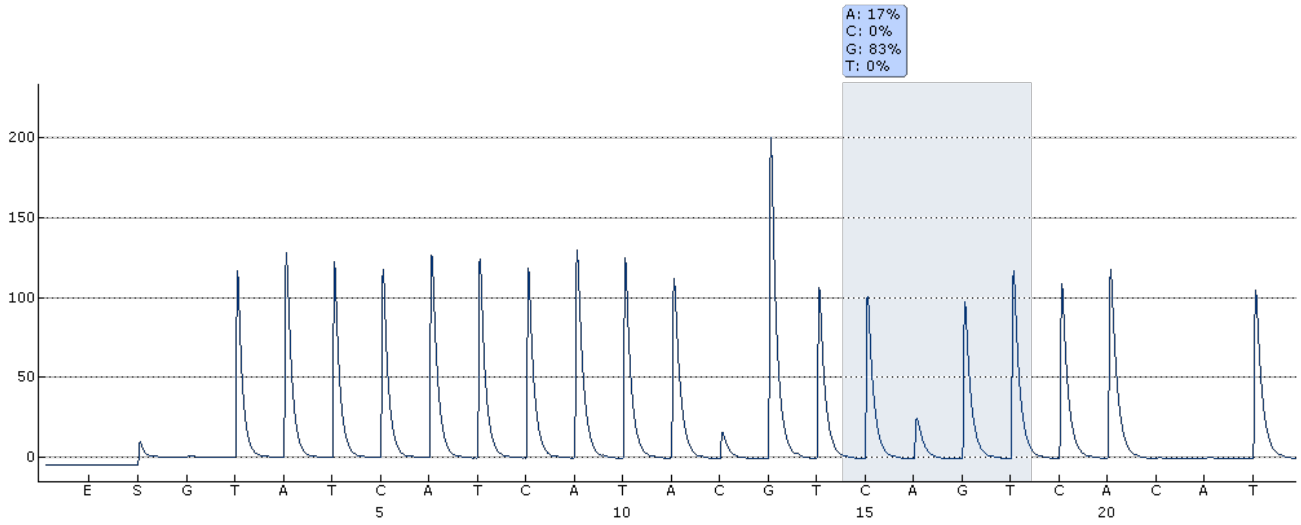

Sequence to analyze:
TATCATCATAGGTCNTCATGCTTAT

Position	1
Quality	Passed
A (%)	17
C (%)	0
G (%)	83
T (%)	0

No warnings.

Well: C1
Assay: IDH1-2
Sample ID: NT2
Note:
Analysis version: 1.0.10

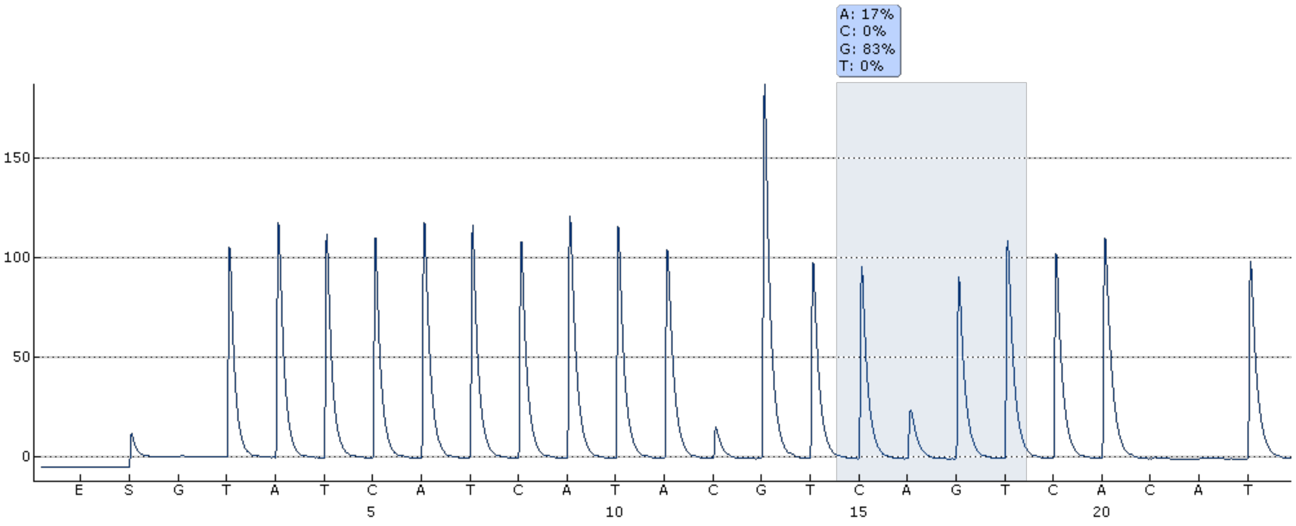

Sequence to analyze:
TATCATCATAGGTCNTCATGCTTAT

Position	1
Quality	Passed
A (%)	17
C (%)	0
G (%)	83
T (%)	0

No warnings.

Well: C2
Assay: IDH1-2
Sample ID: JEG-3
Note:
Analysis version: 1.0.10

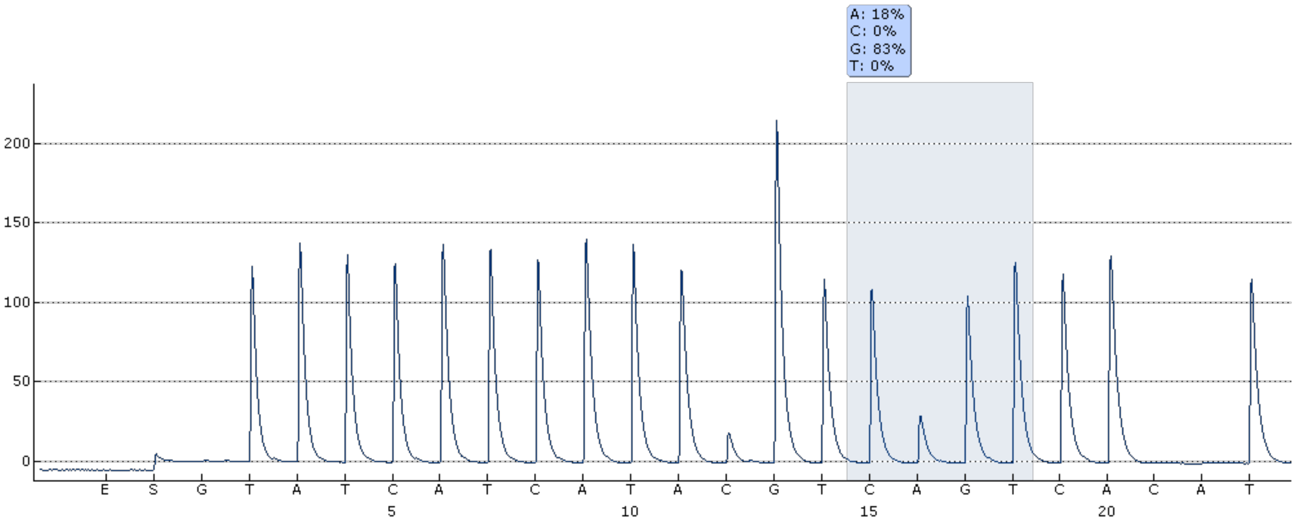

Sequence to analyze:
TATCATCATAGGTCNTCATGCTTAT

Position	1
Quality	Passed
A (%)	18
C (%)	0
G (%)	83
T (%)	0

No warnings.

Well: C3
Assay: IDH1-2
Sample ID: CCT
Note:
Analysis version: 1.0.10

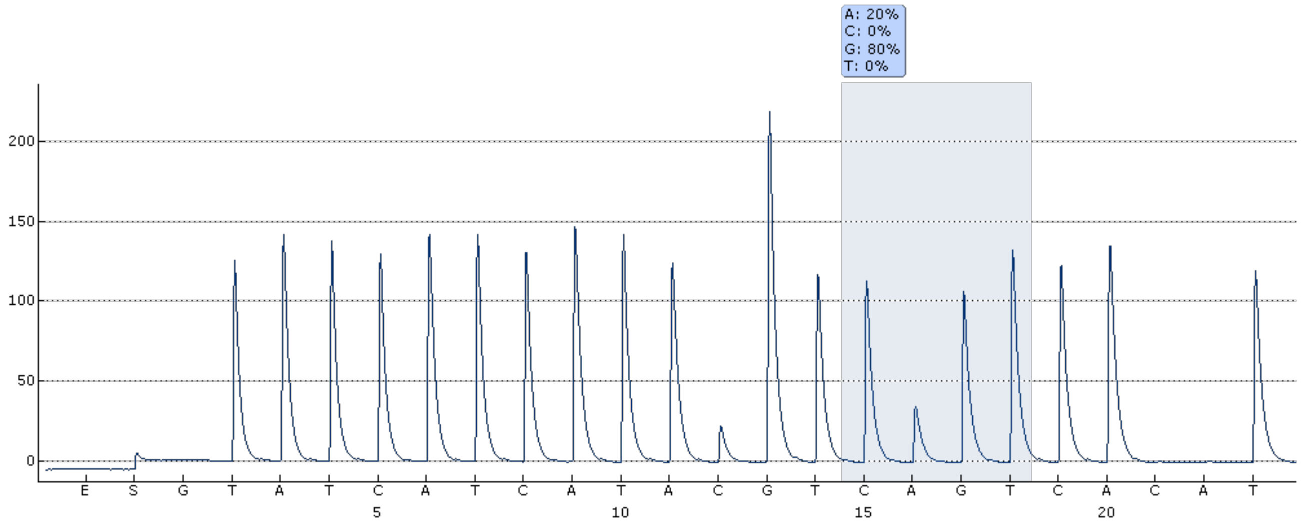

Sequence to analyze:
TATCATCATAGGTCNTCATGCTTAT

Position	1
Quality	Passed
A (%)	20
C (%)	0
G (%)	80
T (%)	0

No warnings.

Well: C4
Assay: IDH1-2
Sample ID: 2102 EP
Note:
Analysis version: 1.0.10

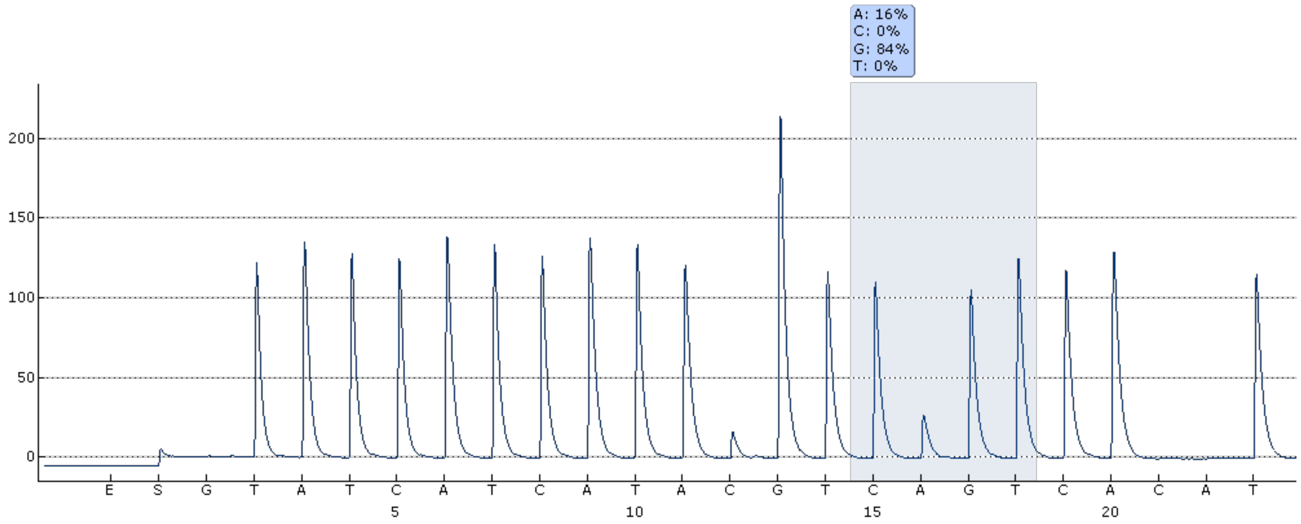

Sequence to analyze:
TATCATCATAGGTCNTCATGCTTAT

Position	1
Quality	Passed
A (%)	16
C (%)	0
G (%)	84
T (%)	0

No warnings.

Well: C5
Assay: IDH1-2
Sample ID: H12
Note:
Analysis version: 1.0.10

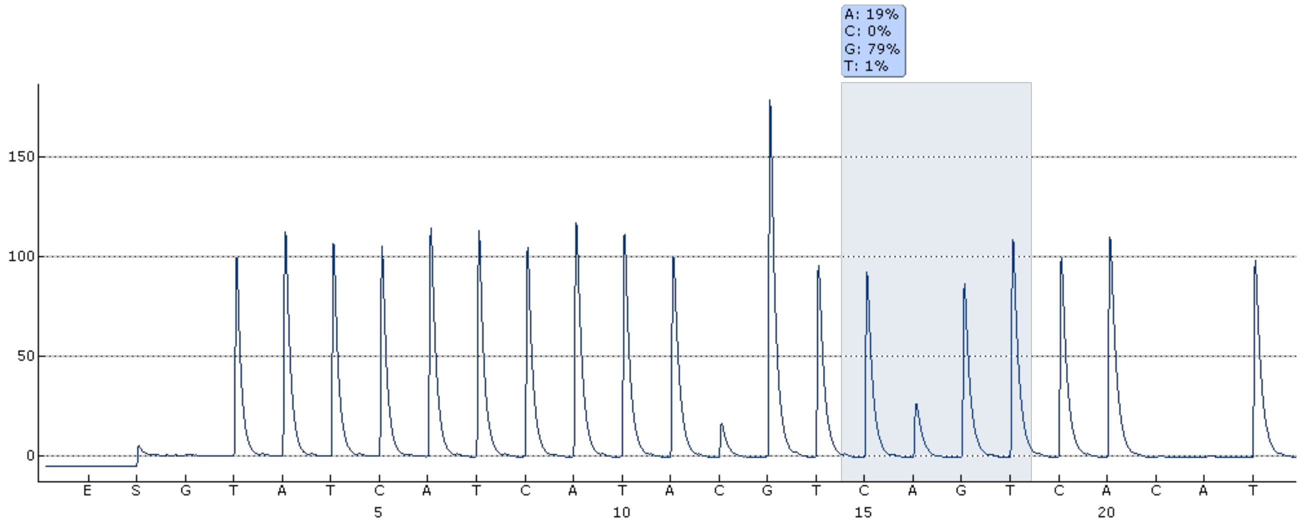

Sequence to analyze:
TATCATCATAGGTCNTCATGCTTAT

Position	1
Quality	Passed
A (%)	19
C (%)	0
G (%)	79
T (%)	1

No warnings.

Well: C6
Assay: IDH1-2
Sample ID: 577 M
Note:
Analysis version: 1.0.10

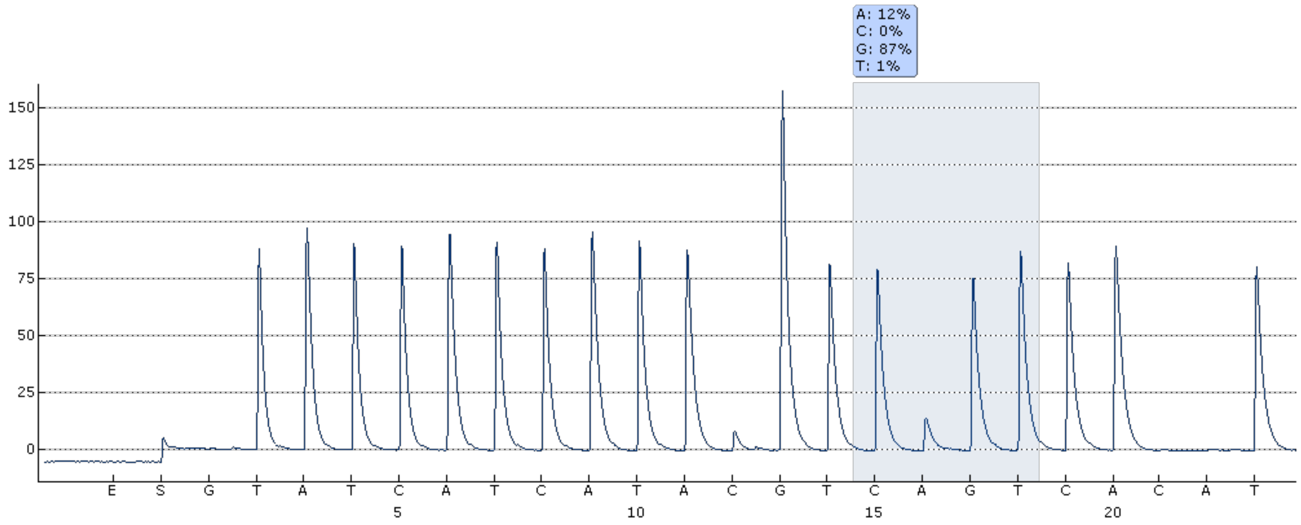

Sequence to analyze:
TATCATCATAGGTCNTCATGCTTAT

Position	1
Quality	Passed
A (%)	12
C (%)	0
G (%)	87
T (%)	1

No warnings.

Well: C7
Assay: IDH1-2
Sample ID: 833 K
Note:
Analysis version: 1.0.10

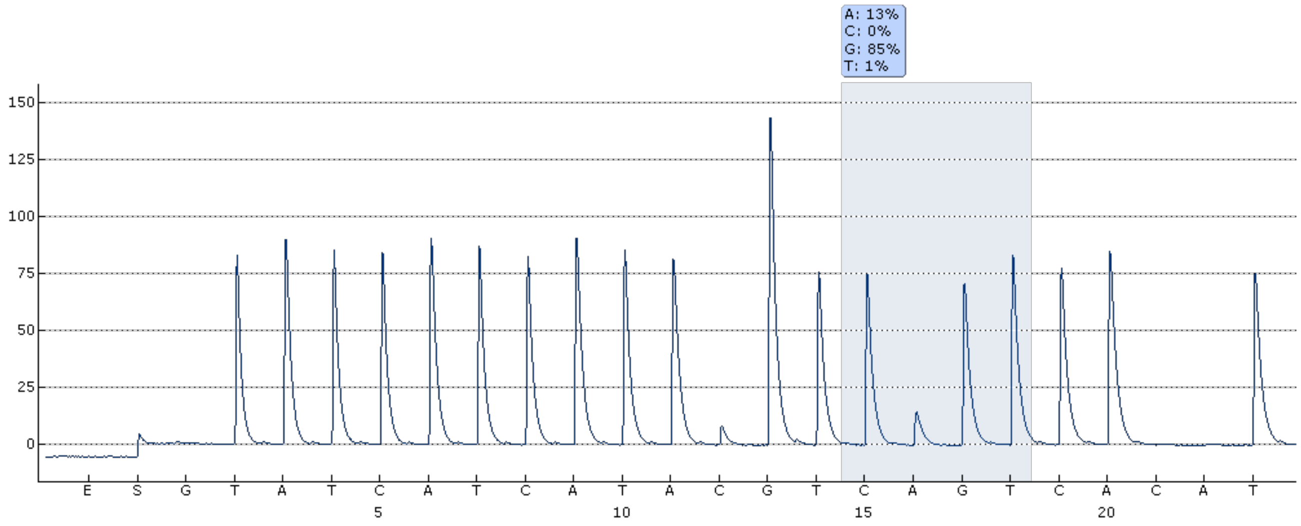

Sequence to analyze:
TATCATCATAGGTCNTCATGCTTAT

Position	1
Quality	Passed
A (%)	13
C (%)	0
G (%)	85
T (%)	1

No warnings.

Well: C8
Assay: IDH1-2
Sample ID: 1411
Note:
Analysis version: 1.0.10

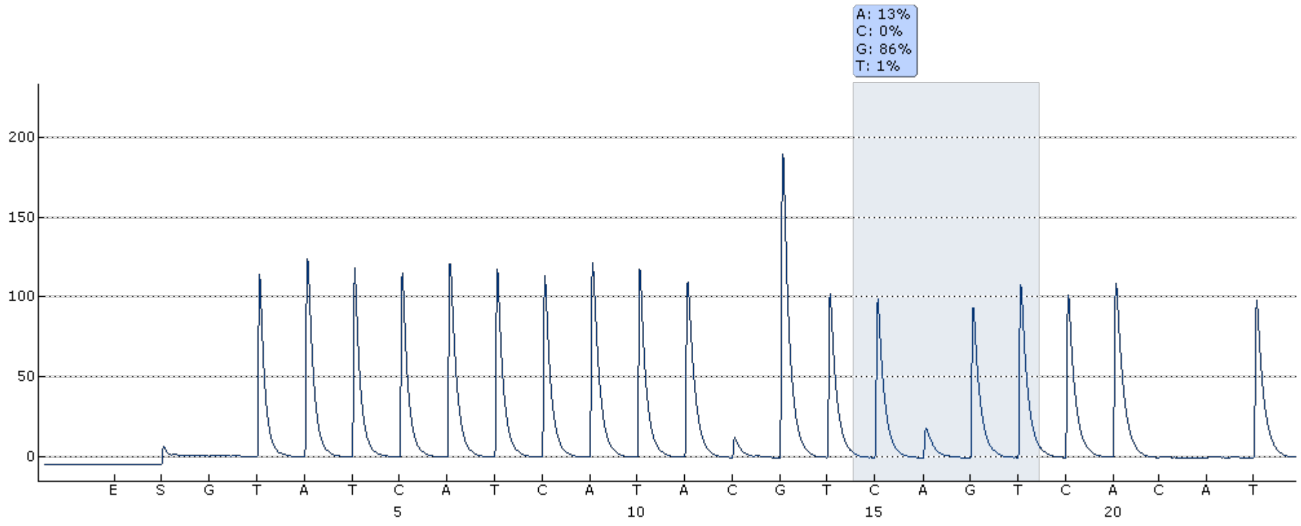

Sequence to analyze:
TATCATCATAGGTCNTCATGCTTAT

Position	1
Quality	Passed
A (%)	13
C (%)	0
G (%)	86
T (%)	1

No warnings.

AQ Full Report

Run Info

Run Name	IDH2 25.06.12
Operator	NP-2342F08B15F7\Andreas Waha
Run Date/Time	25.06.2012 12:11:55
Instrument Name	PyroMark Q24
Serial Number	000019
Instrument Method	PyroMark Q24 Method 001 Rev. A
Plate ID	
Barcode	
Reagent ID	
Run Note	

Run Log

0h 0min 0s	information	Run started
0h 0min 7s	information	Cooler started

Analysis results

Well: A1
Assay: IDH2-1
Sample ID: JAR
Note:
Analysis version: 1.0.10

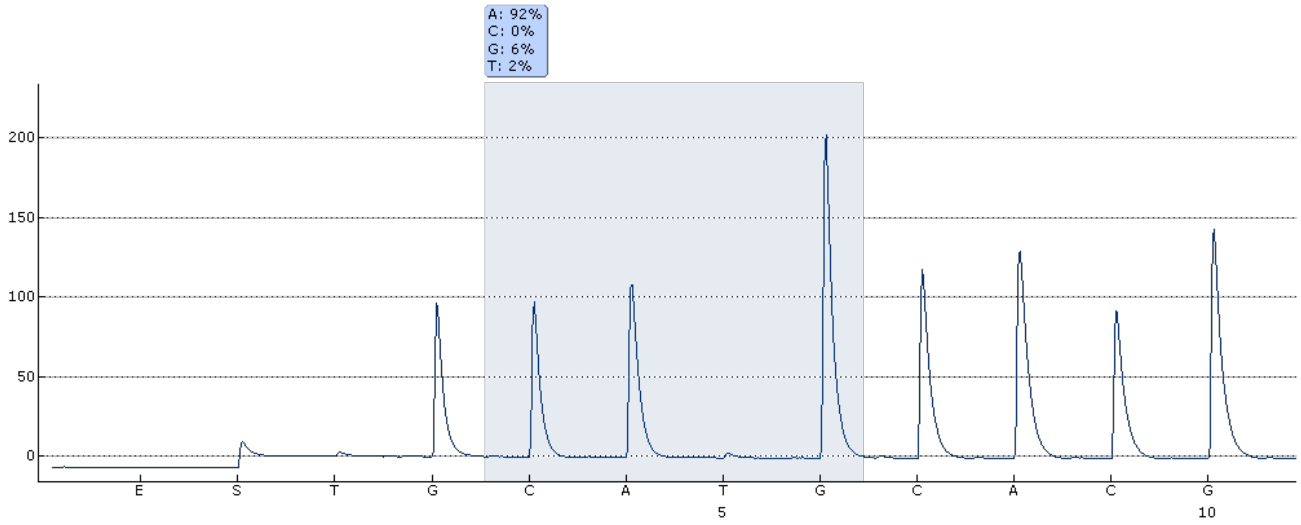

Sequence to analyze:
GCNGGCACGCCCATGGCGACC

Position	1
Quality	Passed
A (%)	92
C (%)	0
G (%)	6
T (%)	2

No warnings.

Well: A2
Assay: IDH2-1
Sample ID: JKT-1
Note:
Analysis version: 1.0.10

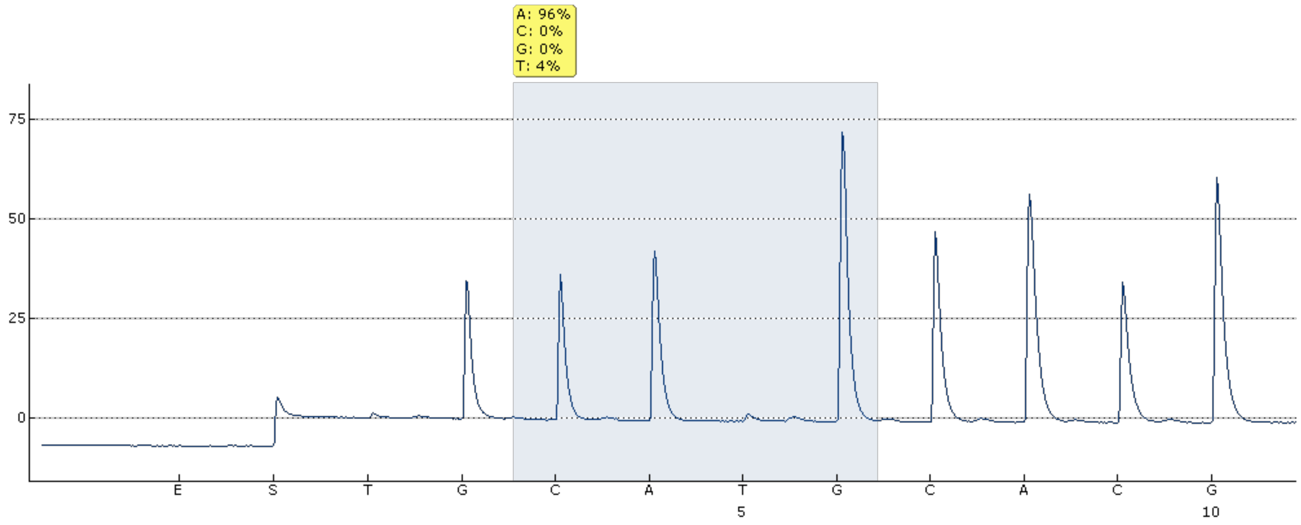

Sequence to analyze:
GCNGGCACGCCCATGGCGACC

Position	1
Quality	Check
A (%)	96
C (%)	0
G (%)	0
T (%)	4

Warnings:
Position 1: Uncertain surrounding reference sequence pattern.

Well: A3
Assay: IDH2-1
Sample ID: NCCIT
Note:
Analysis version: 1.0.10

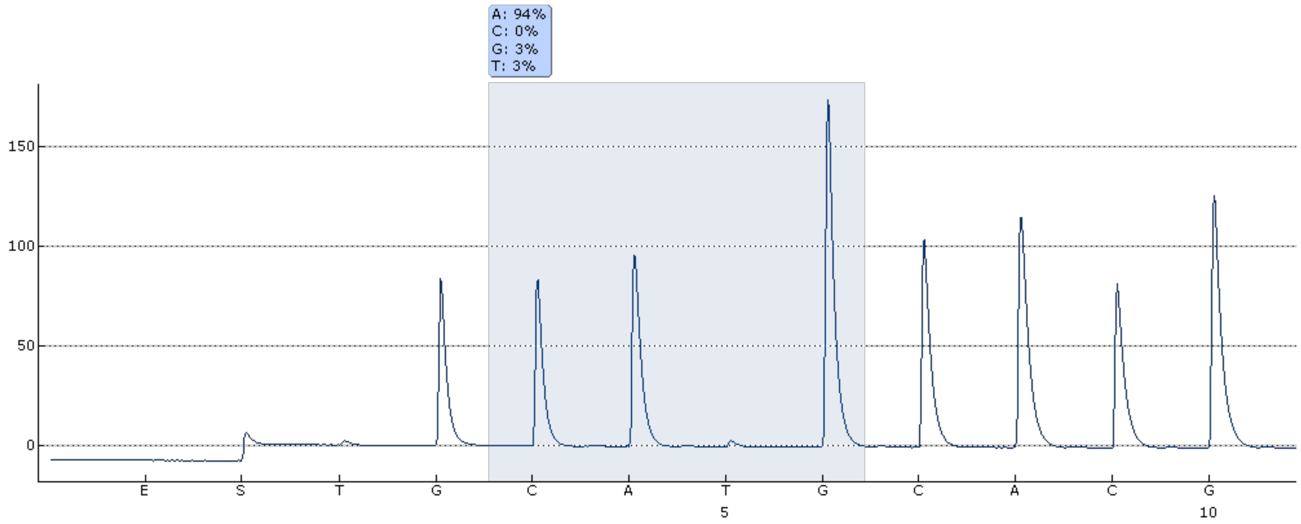

Sequence to analyze:
GCNGGCACGCCCATGGCGACC

Position	1
Quality	Passed
A (%)	94
C (%)	0
G (%)	3
T (%)	3

No warnings.

Well: A4
Assay: IDH2-1
Sample ID: TCam-2
Note:
Analysis version: 1.0.10

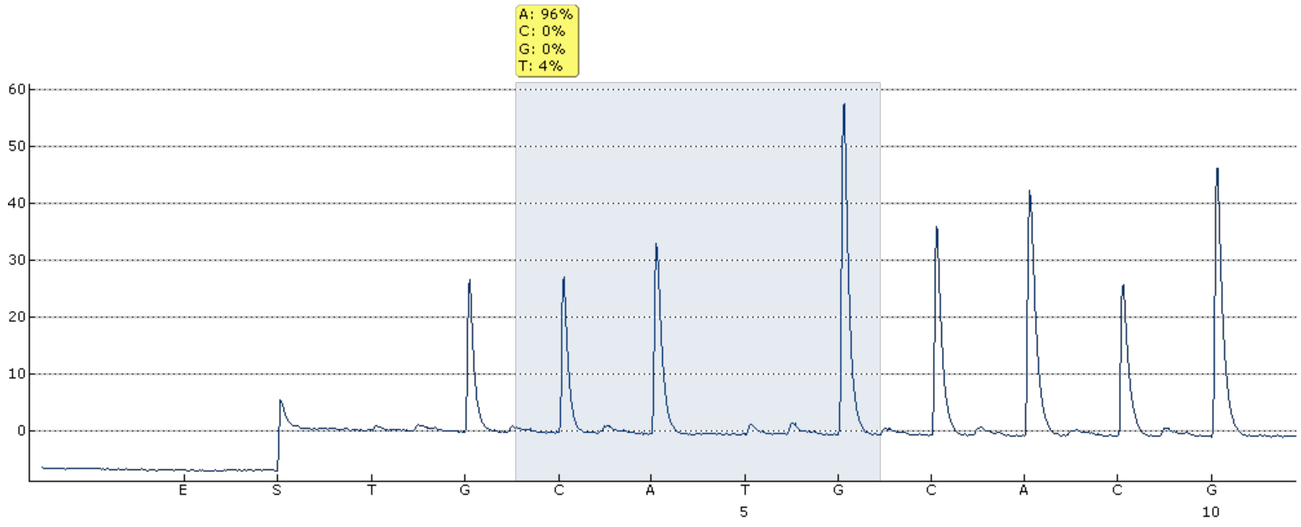

Sequence to analyze:
GCNGGCACGCCCATGGCGACC

Position	1
Quality	Check
A (%)	96
C (%)	0
G (%)	0
T (%)	4

Warnings:
Position 1: Uncertain reference sequence pattern at dispensation: 10.

Well: A5
Assay: IDH2-1
Sample ID: NT2
Note:
Analysis version: 1.0.10

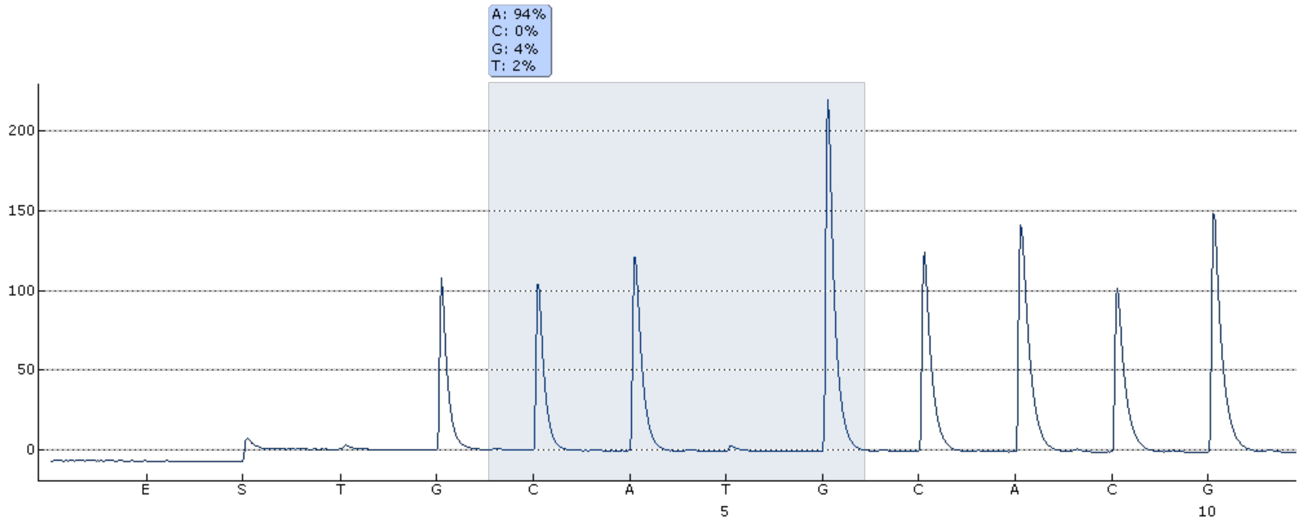

Sequence to analyze:
GCNGGCACGCCCATGGCGACC

Position	1
Quality	Passed
A (%)	94
C (%)	0
G (%)	4
T (%)	2

No warnings.

Well: A6
Assay: IDH2-1
Sample ID: JEG-3
Note:
Analysis version: 1.0.10

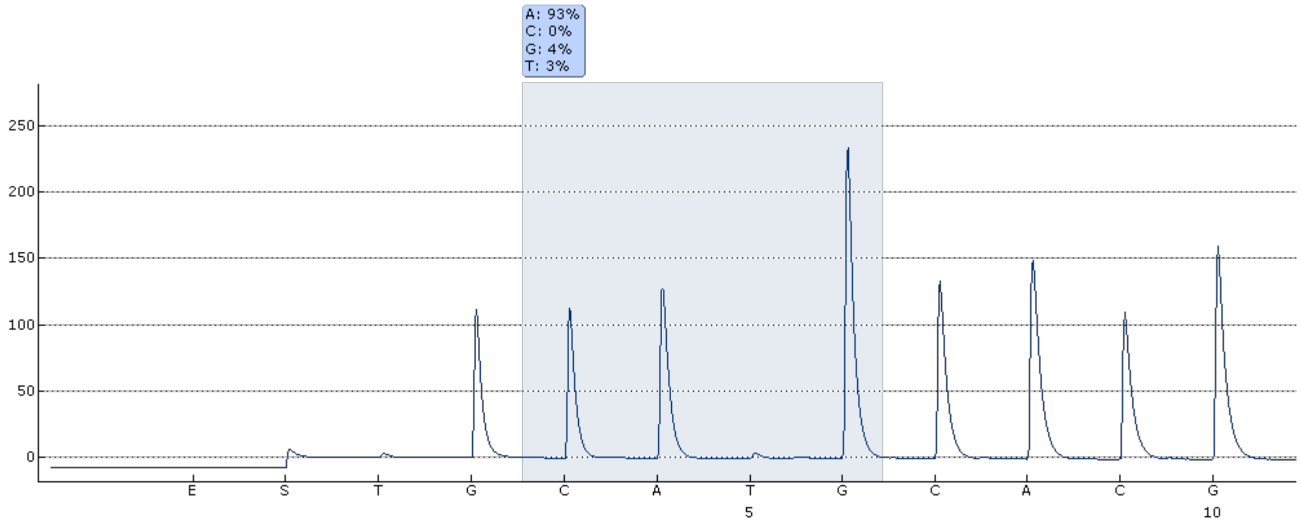

Sequence to analyze:
GCNGGCACGCCCATGGCGACC

Position	1
Quality	Passed
A (%)	93
C (%)	0
G (%)	4
T (%)	3

No warnings.

Well: A7
Assay: IDH2-1
Sample ID: CCT
Note:
Analysis version: 1.0.10

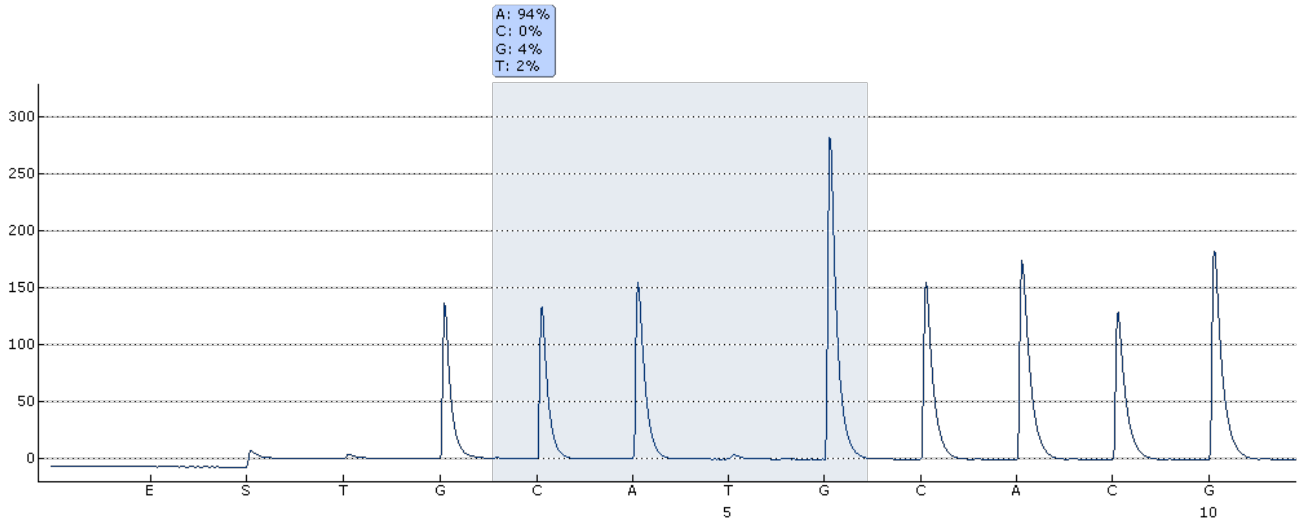

Sequence to analyze:
GCNGGCACGCCCATGGCGACC

Position	1
Quality	Passed
A (%)	94
C (%)	0
G (%)	4
T (%)	2

No warnings.

Well: A8
Assay: IDH2-1
Sample ID: 2102 EP
Note:
Analysis version: 1.0.10

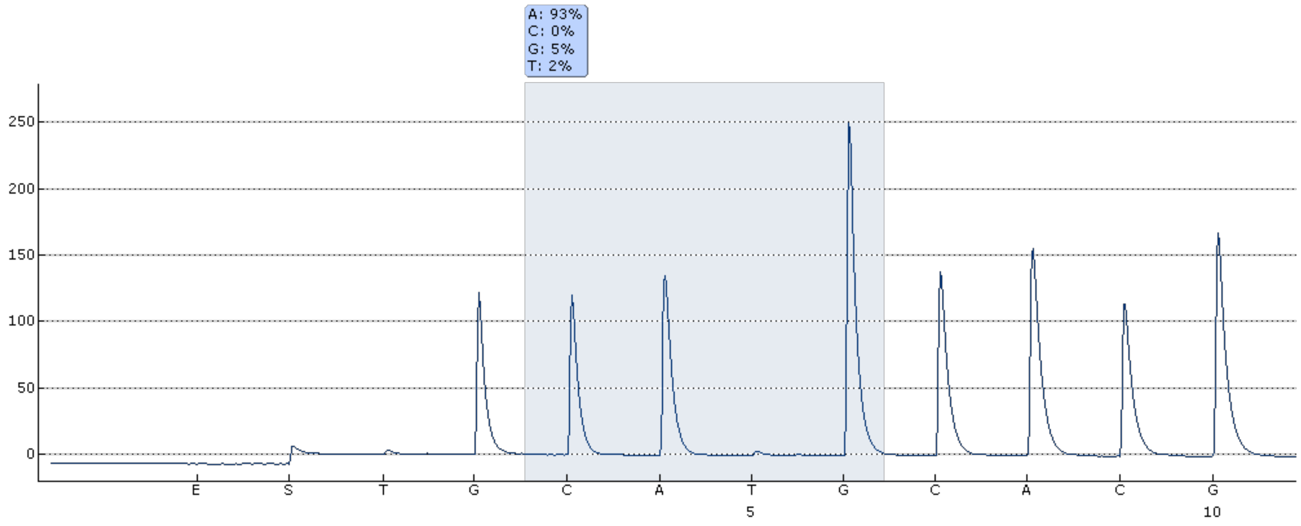

Sequence to analyze:
GCNGGCACGCCCATGGCGACC

Position	1
Quality	Passed
A (%)	93
C (%)	0
G (%)	5
T (%)	2

No warnings.

Well: B1
Assay: IDH2-1
Sample ID: H12
Note:
Analysis version: 1.0.10

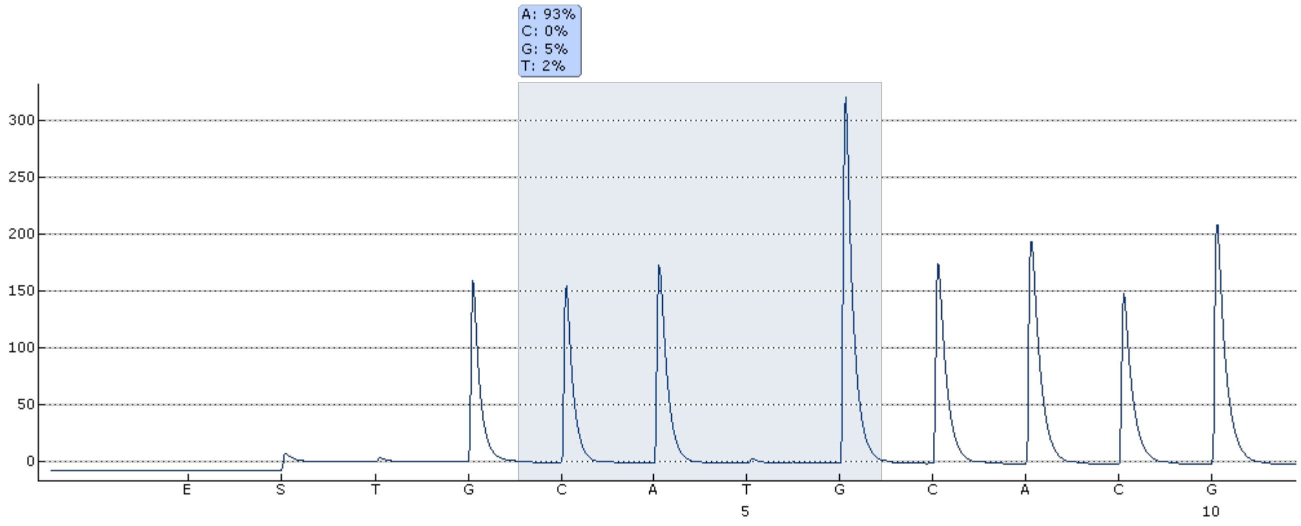

Sequence to analyze:
GCNGGCACGCCCATGGCGACC

Position	1
Quality	Passed
A (%)	93
C (%)	0
G (%)	5
T (%)	2

No warnings.

Well: B2
Assay: IDH2-1
Sample ID: 577 M
Note:
Analysis version: 1.0.10

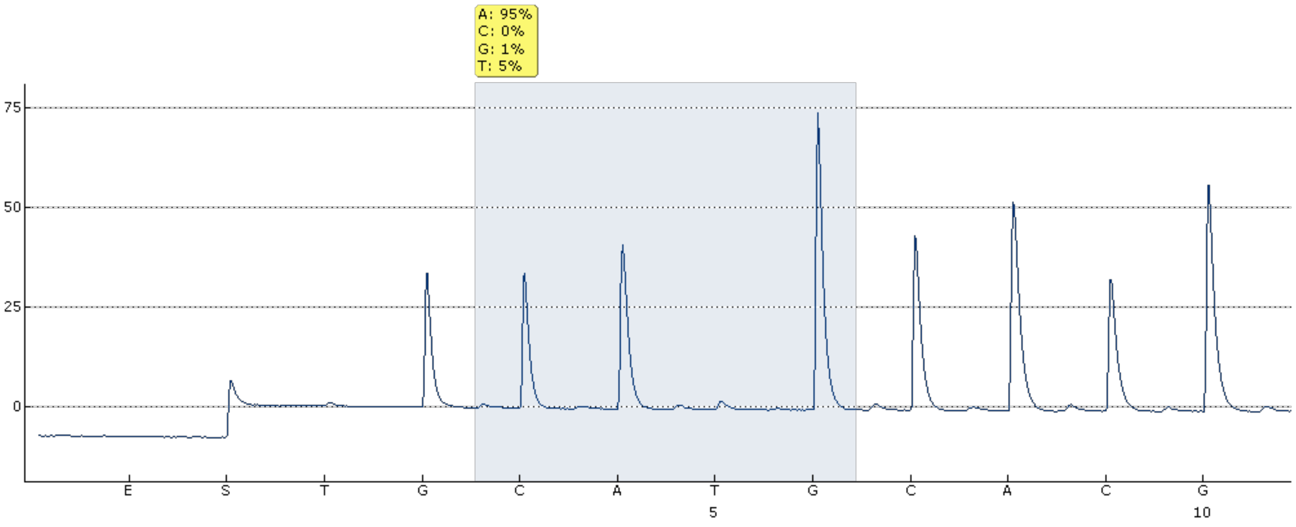

Sequence to analyze:
GCNGGCACGCCCATGGCGACC

Position	1
Quality	Check
A (%)	95
C (%)	0
G (%)	1
T (%)	5

Warnings:
Position 1: Uncertain reference sequence pattern at dispensation: 10.

Well: B3
Assay: IDH2-1
Sample ID: 833 K
Note:
Analysis version: 1.0.10

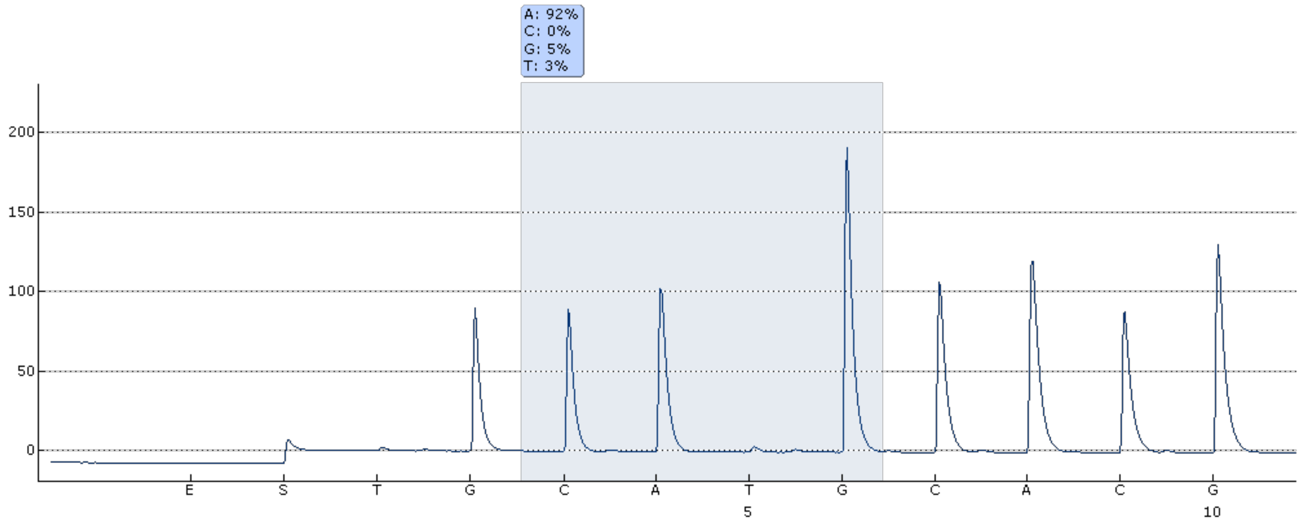

Sequence to analyze:
GCNGGCACGCCCATGGCGACC

Position	1
Quality	Passed
A (%)	92
C (%)	0
G (%)	5
T (%)	3

No warnings.

Well: B4
Assay: IDH2-1
Sample ID: 1411
Note:
Analysis version: 1.0.10

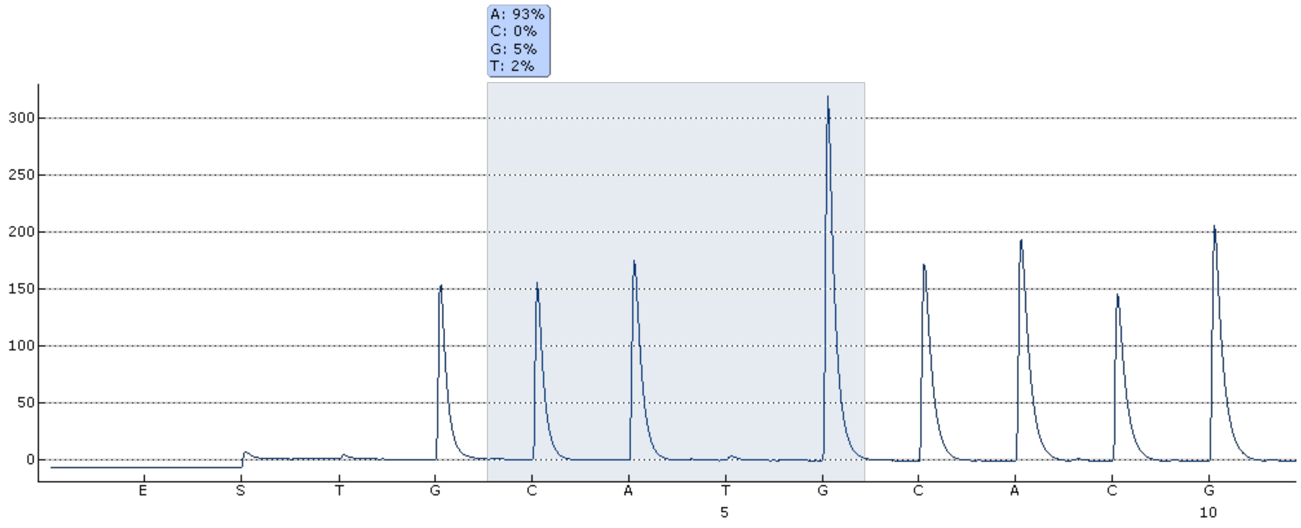

Sequence to analyze:
GCNGGCACGCCCATGGCGACC

Position	1
Quality	Passed
A (%)	93
C (%)	0
G (%)	5
T (%)	2

No warnings.

Well: B5
Assay: IDH2-2
Sample ID: JAR
Note:
Analysis version: 1.0.10

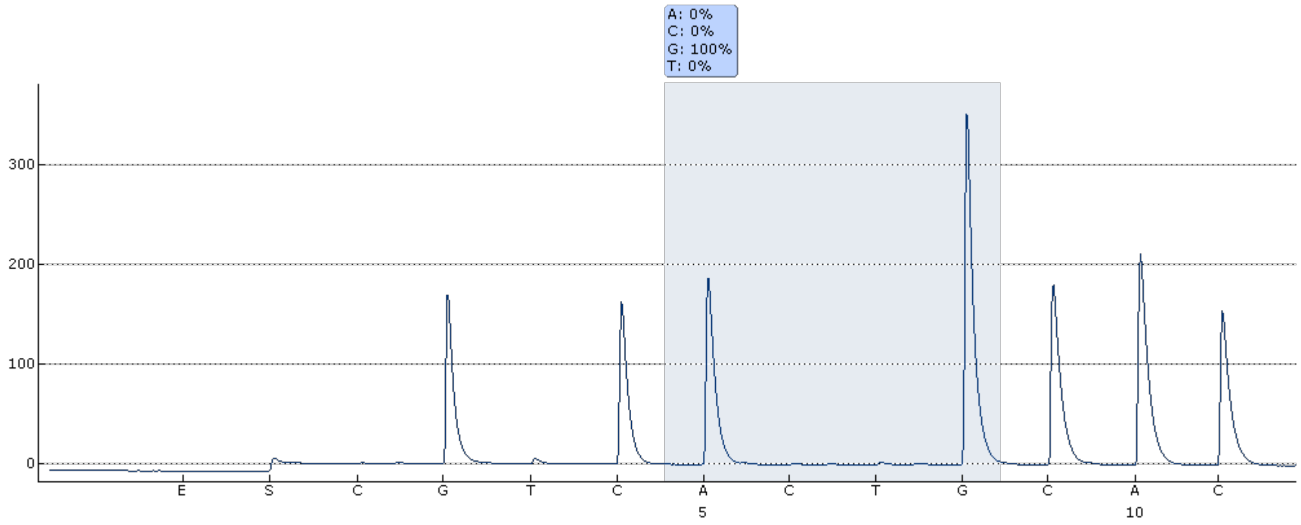

Sequence to analyze:
GCANGCACGCCCATGGCGACC

Position	1
Quality	Passed
A (%)	0
C (%)	0
G (%)	100
T (%)	0

No warnings.

Well: B6
Assay: IDH2-2
Sample ID: JKT-1
Note:
Analysis version: 1.0.10

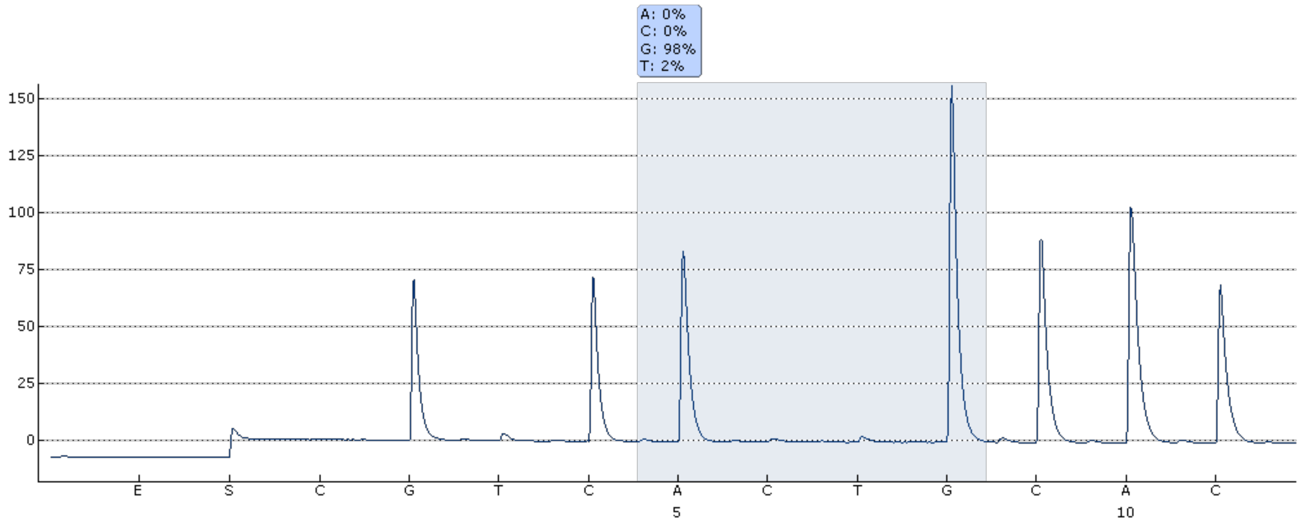

Sequence to analyze:
GCANGCACGCCCATGGCGACC

Position	1
Quality	Passed
A (%)	0
C (%)	0
G (%)	98
T (%)	2

No warnings.

Well: B7
Assay: IDH2-2
Sample ID: NCCIT
Note:
Analysis version: 1.0.10

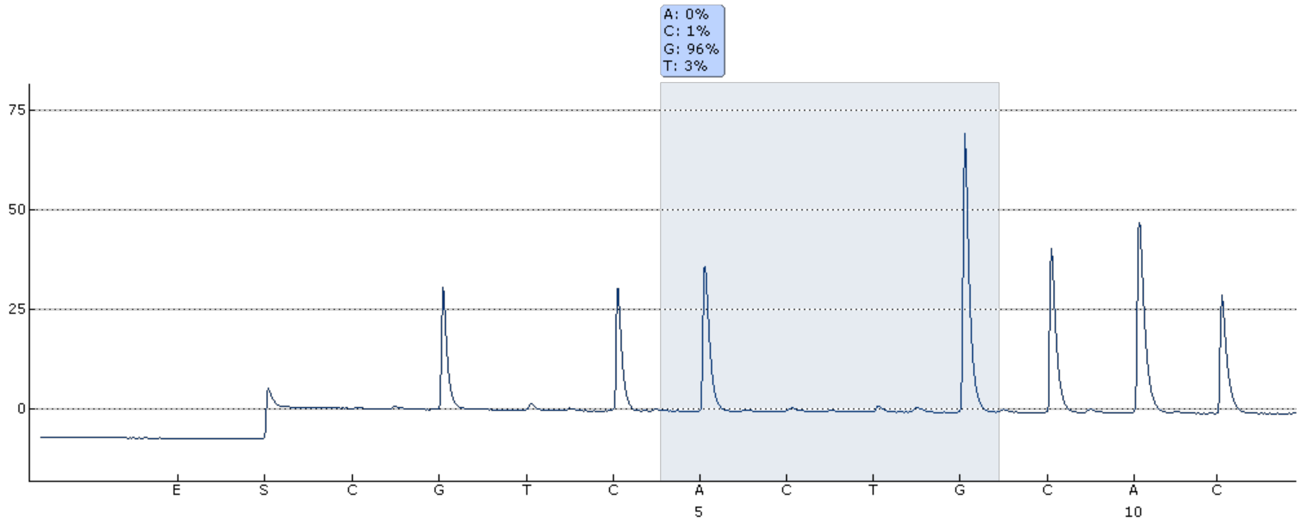

Sequence to analyze:
GCANGCACGCCCATGGCGACC

Position	1
Quality	Passed
A (%)	0
C (%)	1
G (%)	96
T (%)	3

No warnings.

Well: B8
Assay: IDH2-2
Sample ID: TCam-2
Note:
Analysis version: 1.0.10

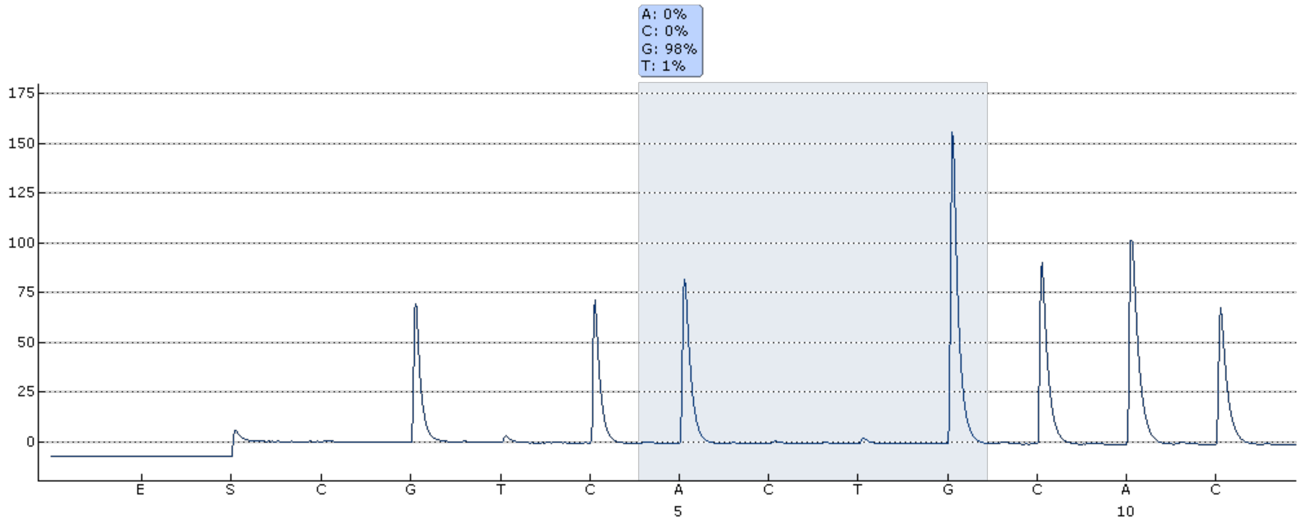

Sequence to analyze:
GCANGCACGCCCATGGCGACC

Position	1
Quality	Passed
A (%)	0
C (%)	0
G (%)	98
T (%)	1

No warnings.

Well: C1
Assay: IDH2-2
Sample ID: NT2
Note:
Analysis version: 1.0.10

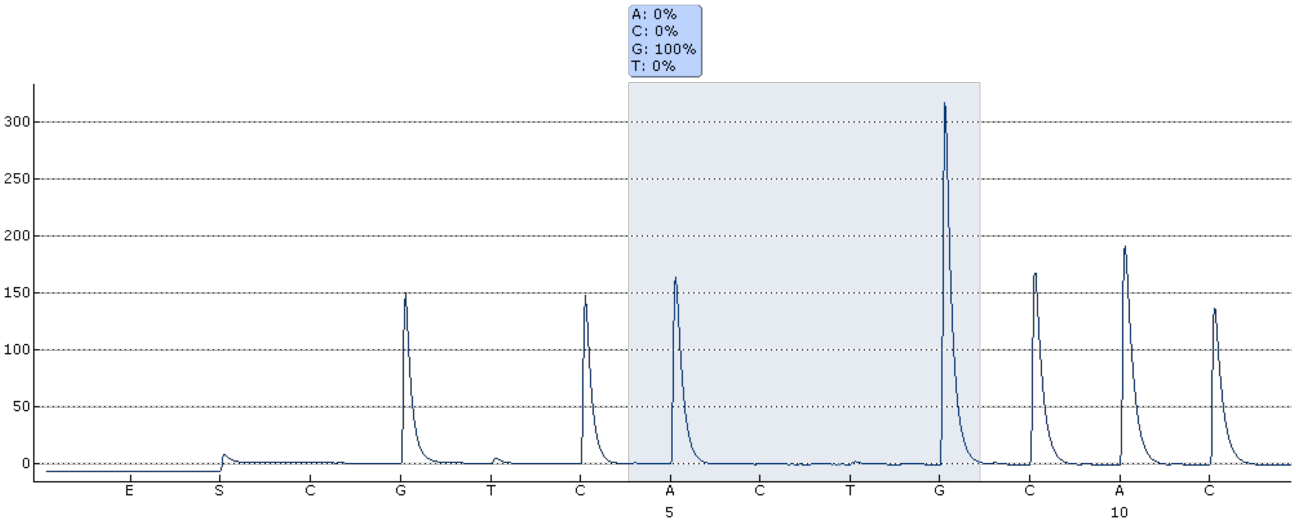

Sequence to analyze:
GCANGCACGCCCATGGCGACC

Position	1
Quality	Passed
A (%)	0
C (%)	0
G (%)	100
T (%)	0

No warnings.

Well: C2
Assay: IDH2-2
Sample ID: JEG-3
Note:
Analysis version: 1.0.10

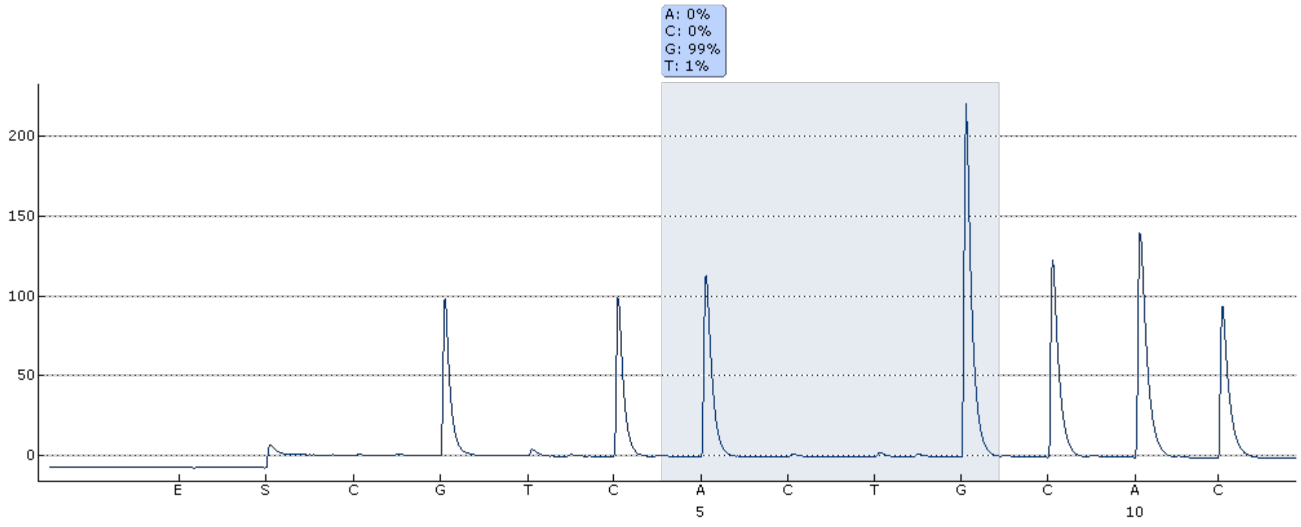

Sequence to analyze:
GCANGCACGCCCATGGCGACC

Position	1
Quality	Passed
A (%)	0
C (%)	0
G (%)	99
T (%)	1

No warnings.

Well: C3
Assay: IDH2-2
Sample ID: CCT
Note:
Analysis version: 1.0.10

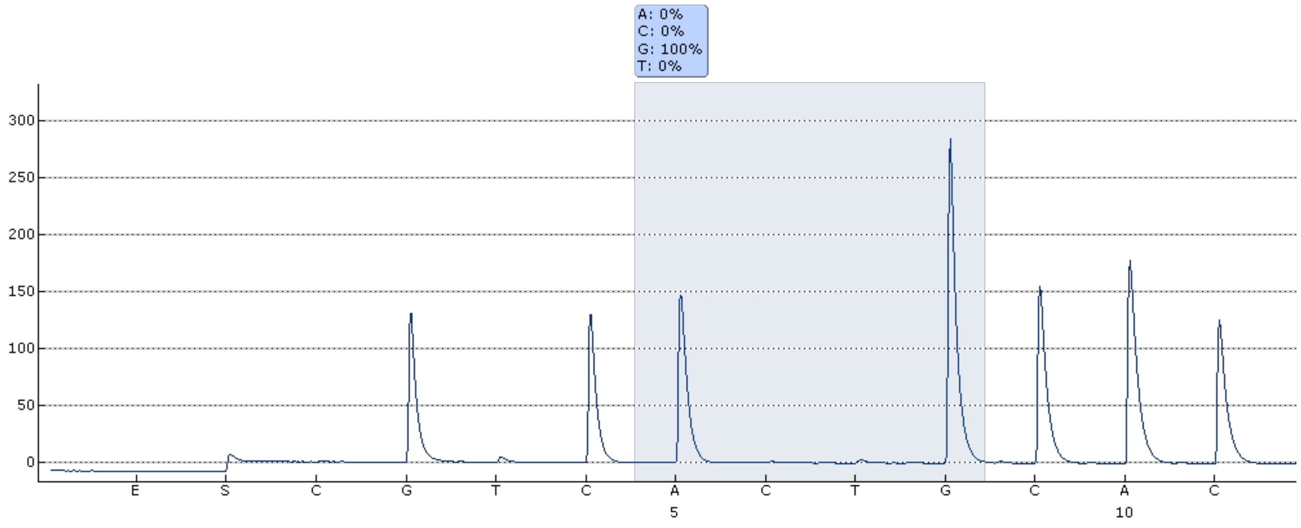

Sequence to analyze:
GCANGCACGCCCATGGCGACC

Position	1
Quality	Passed
A (%)	0
C (%)	0
G (%)	100
T (%)	0

No warnings.

Well: C4
Assay: IDH2-2
Sample ID: 2102 EP
Note:
Analysis version: 1.0.10

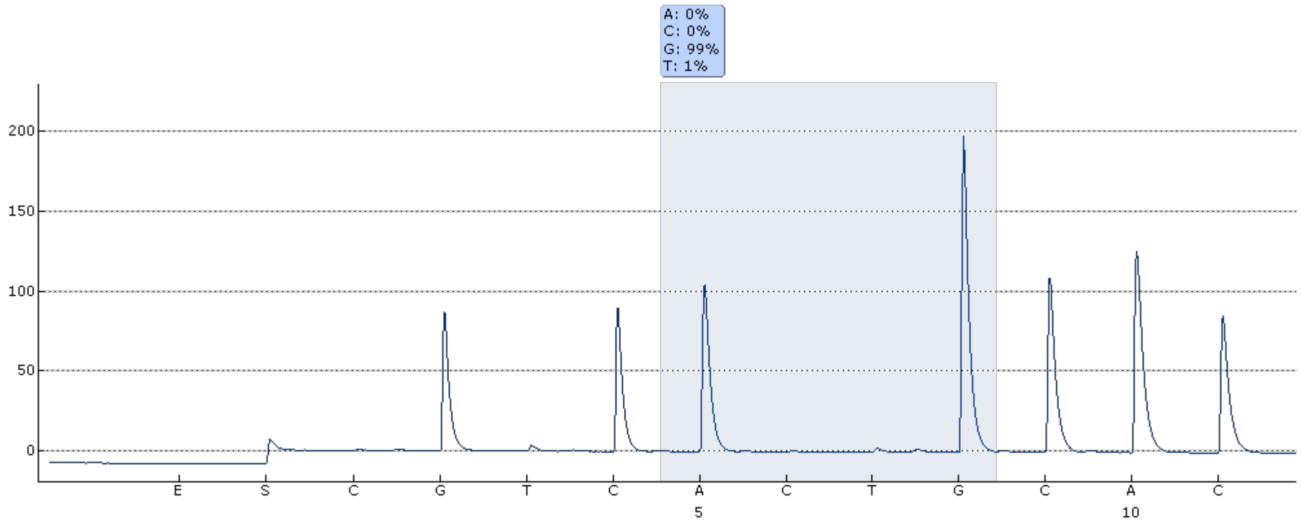

Sequence to analyze:
GCANGCACGCCCATGGCGACC

Position	1
Quality	Passed
A (%)	0
C (%)	0
G (%)	99
T (%)	1

No warnings.

Well: C5
Assay: IDH2-2
Sample ID: H12
Note:
Analysis version: 1.0.10

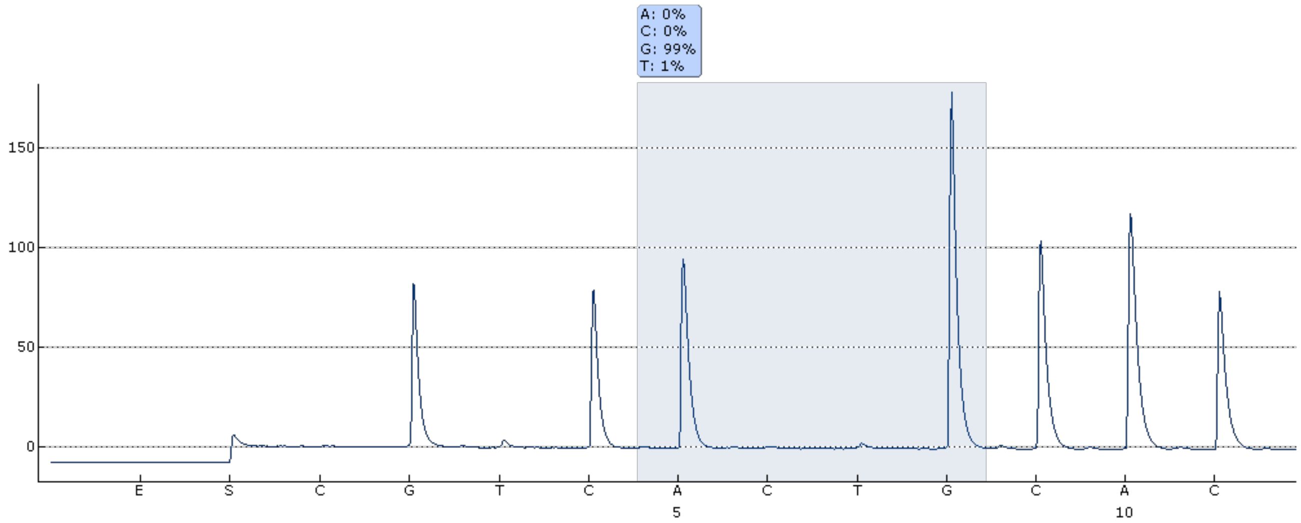

Sequence to analyze:
GCANGCACGCCCATGGCGACC

Position	1
Quality	Passed
A (%)	0
C (%)	0
G (%)	99
T (%)	1

No warnings.

Well: C6
Assay: IDH2-2
Sample ID: 577 M
Note:
Analysis version: 1.0.10

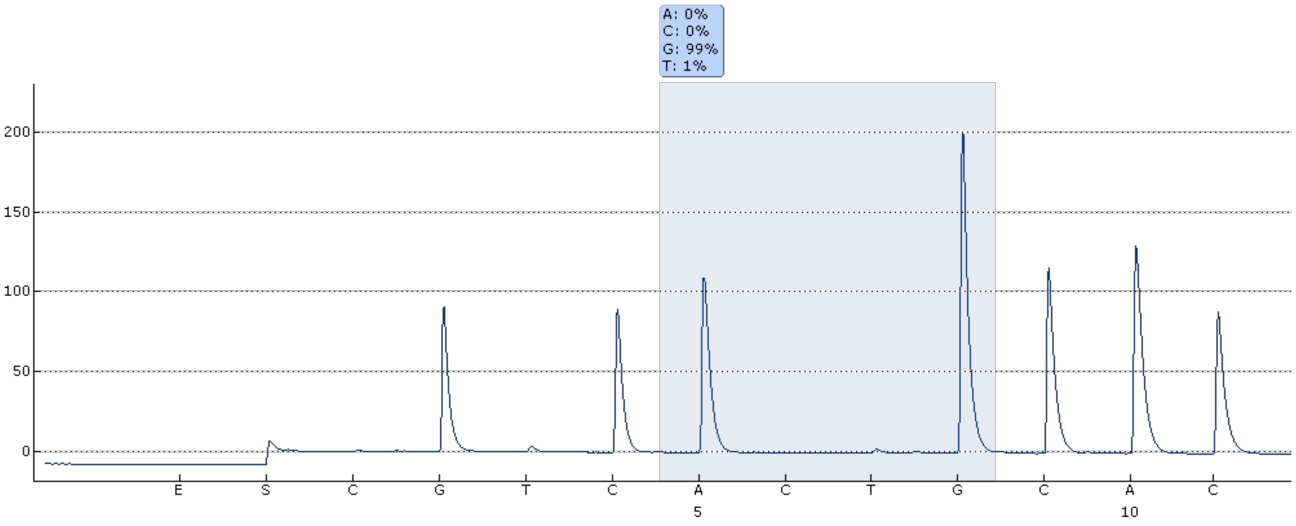

Sequence to analyze:
GCANGCACGCCCATGGCGACC

Position	1
Quality	Passed
A (%)	0
C (%)	0
G (%)	99
T (%)	1

No warnings.

Well: C7
Assay: IDH2-2
Sample ID: 833 K
Note:
Analysis version: 1.0.10

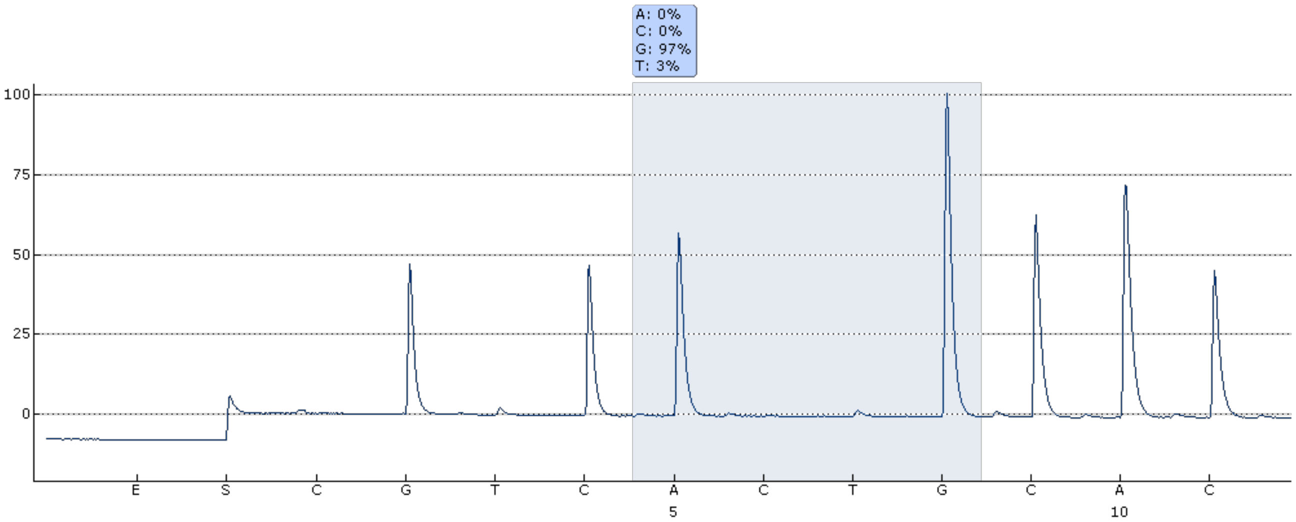

Sequence to analyze:
GCANGCACGCCCATGGCGACC

Position	1
Quality	Passed
A (%)	0
C (%)	0
G (%)	97
T (%)	3

No warnings.

Well: C8
Assay: IDH2-2
Sample ID: 1411
Note:
Analysis version: 1.0.10

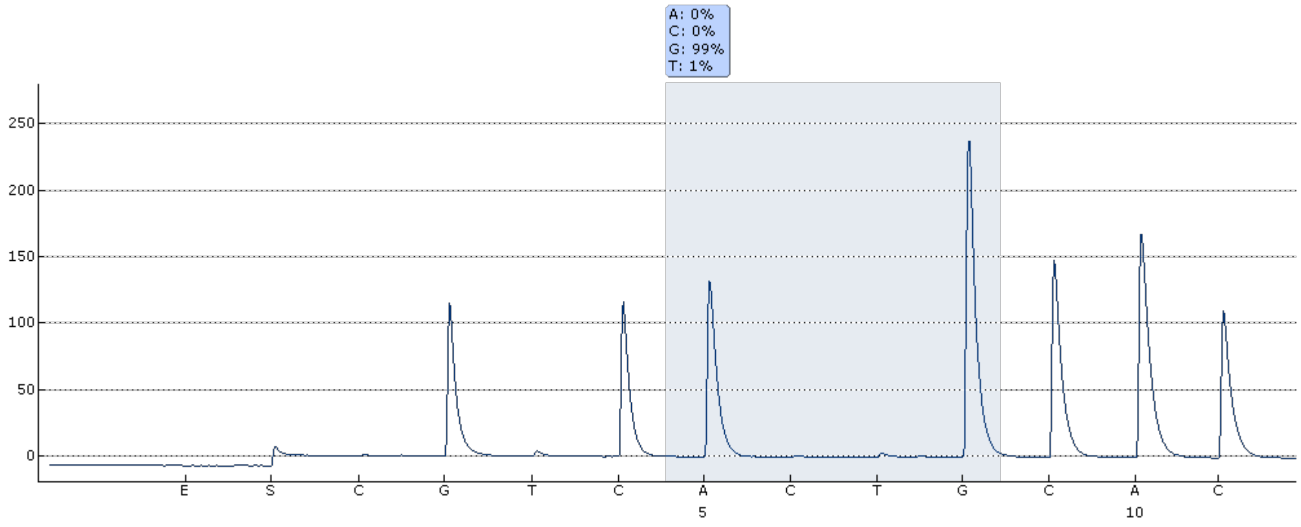

Sequence to analyze:
GCANGCACGCCCATGGCGACC

Position	1
Quality	Passed
A (%)	0
C (%)	0
G (%)	99
T (%)	1

No warnings.

AQ Full Report

Run Info

Run Name	IDH1+IDH2 25.06.12
Operator	NP-2342F08B15F7\Andreas Waha
Run Date/Time	25.06.2012 12:33:49
Instrument Name	PyroMark Q24
Serial Number	000019
Instrument Method	PyroMark Q24 Method 001 Rev. A
Plate ID	
Barcode	
Reagent ID	
Run Note	

Run Log

0h 0min 0s	information	Run started
0h 0min 7s	information	Cooler started

Analysis results

Well: A1
Assay: IDH1-1
Sample ID: 1777
Note:
Analysis version: 1.0.10

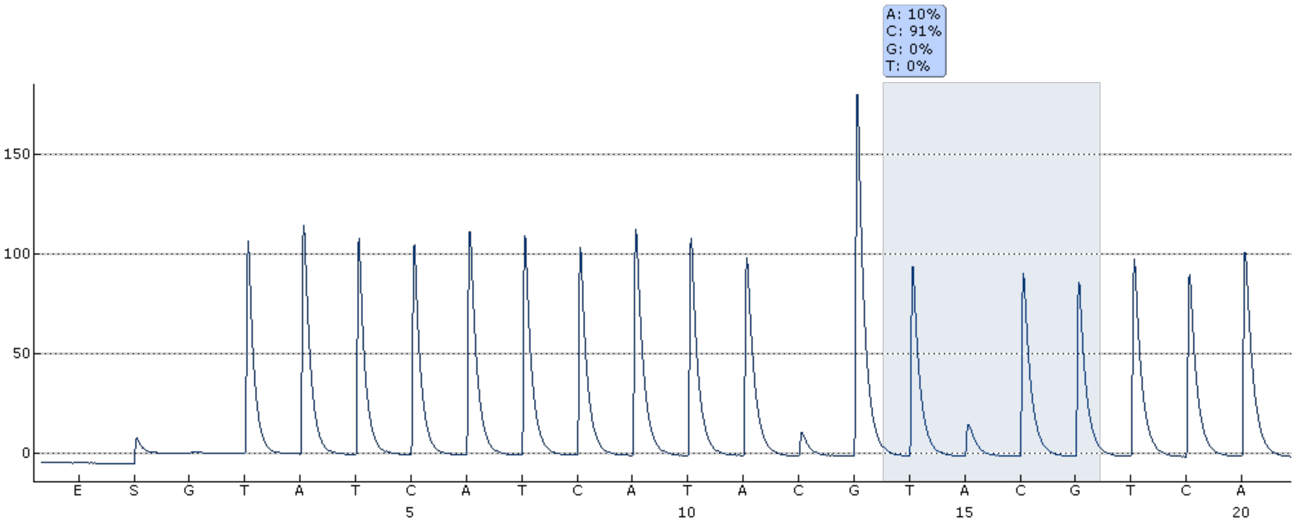

Sequence to analyze:
TATCATCATAGGTNGTCATGC

Position	1
Quality	Passed
A (%)	10
C (%)	91
G (%)	0
T (%)	0

No warnings.

Well: A2
Assay: IDH1-2
Sample ID: 1777
Note:
Analysis version: 1.0.10

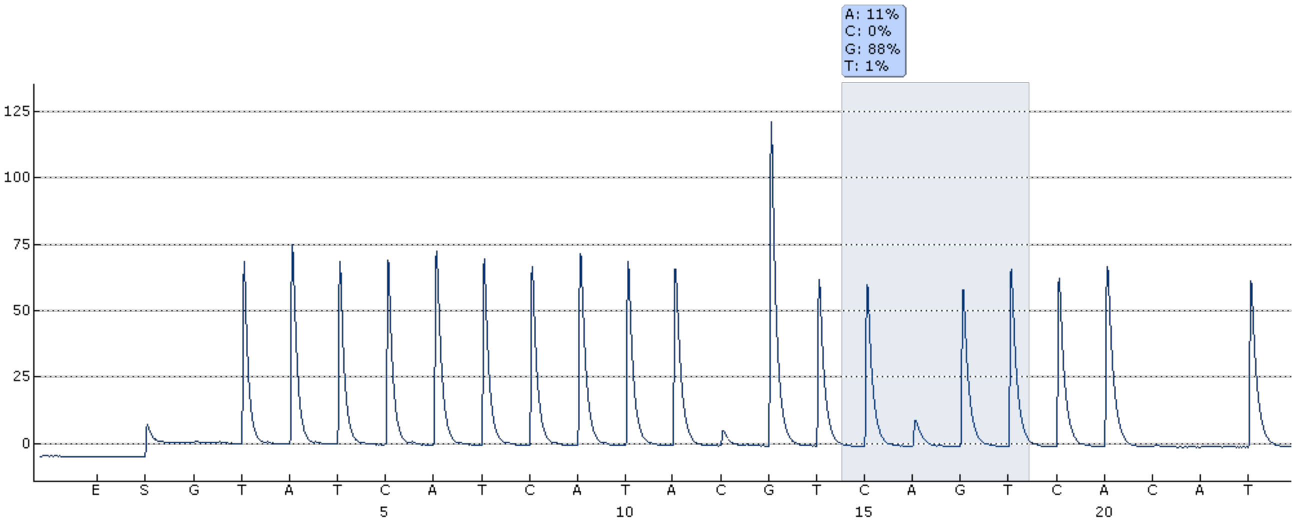

Sequence to analyze:
TATCATCATAGGTCNTCATGCTTAT

Position	1
Quality	Passed
A (%)	11
C (%)	0
G (%)	88
T (%)	1

No warnings.

Well: A3
Assay: IDH2-1
Sample ID: 1777
Note:
Analysis version: 1.0.10

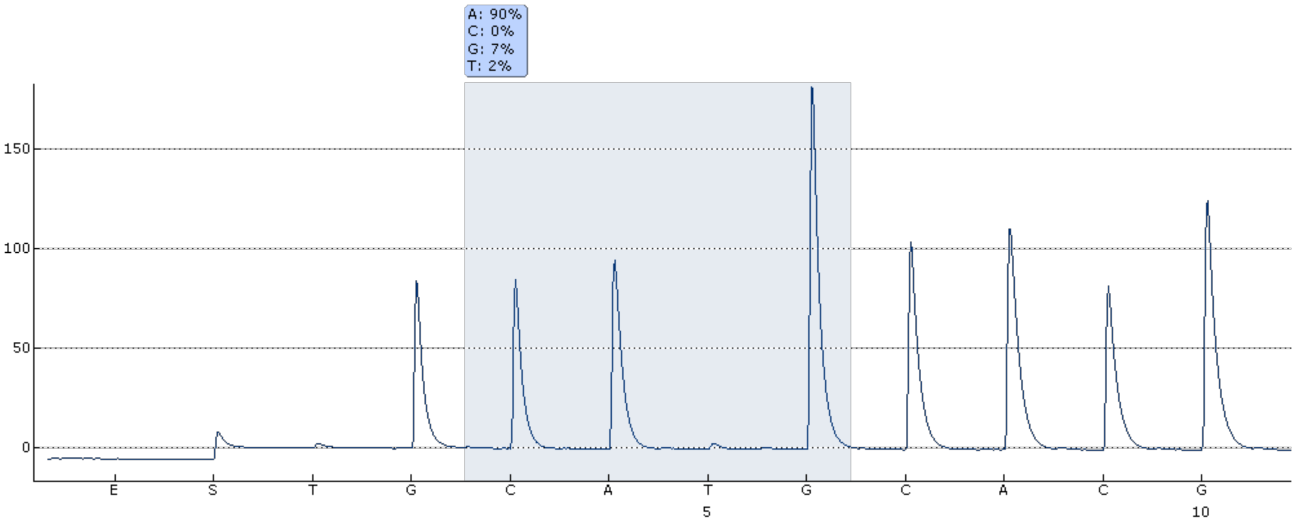

Sequence to analyze:
GCNGGCACGCCCATGGCGACC

Position	1
Quality	Passed
A (%)	90
C (%)	0
G (%)	7
T (%)	2

No warnings.

Well: A4
Assay: IDH2-2
Sample ID: 1777
Note:
Analysis version: 1.0.10

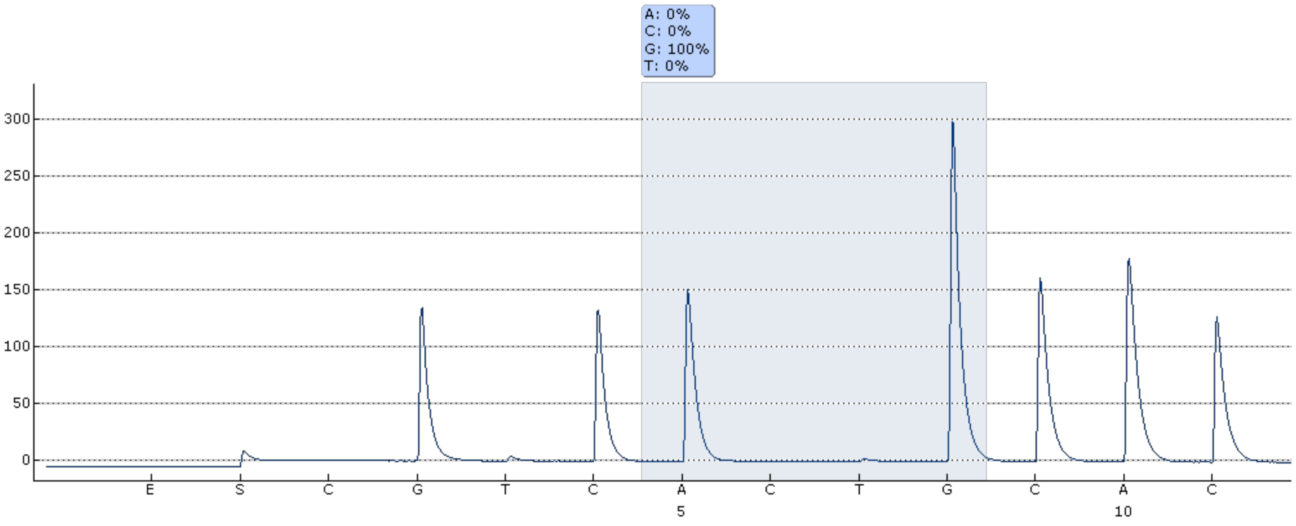

Sequence to analyze:
GCANGCACGCCCATGGCGACC

Position	1
Quality	Passed
A (%)	0
C (%)	0
G (%)	100
T (%)	0

No warnings.
